# Lung dysbiosis disrupts an FFAR2-mediated innate immune circuit against *Klebsiella pneumoniae*

**DOI:** 10.7150/thno.131136

**Published:** 2026-03-12

**Authors:** Ting-Chieh Huang, Jheng-Syuan Shao, Alan Chuan-Ying Lai, Ko-Chien Wu, Da-Fu Lin, Ya-Jen Chang

**Affiliations:** 1 Institute of Biomedical Sciences, Academia Sinica, Taipei, Taiwan, R.O.C.; 2 Graduate Institute of Microbiology and Immunology, National Defense Medical University, Taipei, Taiwan, R.O.C.; 3 College of Public Health, National Defense Medical University, Taipei, Taiwan, R.O.C.; 4 Graduate Institute of Medicine, College of Medicine, Kaohsiung Medical University, Kaohsiung, Taiwan, R.O.C.; 5 Institute of Translational Medicine and New Drug Development, China Medical University, Taichung, Taiwan, R.O.C.

**Keywords:** lung microbiota, SCFA-FFAR2, alveolar macrophage, γδ T cell, *K. pneumoniae*

## Abstract

**Rationale:**

Pneumonia is a leading infectious disease, with Gram-negative bacteria such as *K. pneumoniae* posing serious clinical threats. Host defense against *K. pneumoniae* lung infection largely mediated by innate immune responses. Although gut microbiota has been shown to influence lung immunity via the gut-lung axis, the contribution of lung microbiota remains unclear. This study investigates the role of lung microbiota in *K. pneumoniae* lung infection, aiming to elucidate its functional significance in shaping pulmonary immune responses and susceptibility to bacterial pneumonia.

**Methods:**

Using a vancomycin-induced lung dysbiosis mouse model, we profiled lung bacterial composition by 16S rRNA sequencing and quantified short-chain fatty acid (SCFA) levels in bronchoalveolar lavage fluid (BALF) using liquid chromatography-mass spectrometry (LC-MS). Mice were subsequently challenged with* K. pneumoniae* or LPS via intranasal administration to establish lung infection. We further performed bulk RNA sequencing and reanalyzed single-cell RNA sequencing datasets to dissect microbiota-immune interactions in the lung. Cellular assays, cytokine profiling, gene expression analysis, acetate supplementation, and conditional knockout mice were used to further elucidate the underlying mechanisms.

**Result:**

Here, we show that mice with lung dysbiosis are more susceptible to *K. pneumoniae*, exhibiting reduced IL-17A production and impaired IL-17A⁺ γδ T cell activation due to diminished IL-1β secretion from alveolar macrophages (AMs) with restrained NF-κB and GPR signaling. Additionally, lung dysbiosis decreases SCFA levels in the lung, while FFAR2, a primary SCFA receptor, is predominantly expressed in AMs. Acetate, the major ligand for FFAR2, enhances IL-1β production in AMs and restores dysbiosis-suppressed immune responses in an FFAR2-dependent manner. Moreover, *Ffar2*^fl/fl^*LysM*^cre/+^ mice display compromised resistance to *K. pneumoniae*, and FFAR2 is likewise enriched in human AMs, underscoring translational relevance.

**Conclusion:**

Lung microbiota coordinates AM and γδ T cell activation against *K. pneumoniae* via SCFA-FFAR2 axis, a protective network disrupted by lung dysbiosis. These findings highlight the therapeutic potential of SCFA-FFAR2 targeting.

## Introduction

Bacterial lung infections range from mild illnesses like bronchitis to severe diseases such as pneumonia, tuberculosis and lung abscess. Pneumonia is one of the leading causes of illness and mortality globally, with Gram-negative bacteria being a major cause of severe pneumonia 1. Among these, *Klebsiella pneumoniae* (*K. pneumoniae*) represents a serious global health threat, especially in healthcare settings where antibiotic use and immunocompromised individuals are common. Alarmingly, the rising multidrug resistance has severely limited treatment options, leading to *K. pneumoniae* being recognized as a critical public health concern 2, 3.

Host defense against *K. pneumoniae* lung infection primarily relies on innate immune responses, wherein Toll-like receptor 4 (TLR4)-induced IL-1β promotes IL-17A production through macrophages-innate lymphocytes interactions, thereby regulating epithelial barrier integrity and neutrophil recruitment to enhance early stage of host defense and bacterial clearance 4-6. Previous studies have highlighted the essential role of TLR4 in macrophage-mediated defense against *K. pneumoniae*. TLR4-deficient mice display impaired inflammatory responses, increased bacterial loads, and higher mortality following infection 7-9, showing that lipopolysaccharide (LPS) of *K. pneumoniae*, as a TLR4 ligand, is essential for activating host immune response against infection. Moreover, mice deficient in IL-17A or IL-17RA exhibit impaired host defense against *K. pneumoniae*, characterized by reduced neutrophil recruitment, worsened epithelial damage, and increased bacterial burden in the lung 10-13, indicating that TLR4- as well as IL-1β- and IL17A-associated cytokine signaling are important for host defense against *K. pneumoniae* infection.

In the lung, alveolar macrophages (AMs) are the resident immune cells and serve as the first line of defense against invading pathogens. When bacterial components engage the pattern recognition receptors (PRRs) on AMs, pro-inflammatory cytokines, such as IL-1β and IL-23, are produced, activating innate lymphocytes and subsequent immune responses against infection 14-19. Lung-resident innate lymphocytes, such as ILC3s and γδ T cells, are activated by pro-inflammatory cytokines and secrete IL-17A and IL-22, thereby contributing to neutrophil-mediated host defense and orchestrating mucosal barrier integrity to protect against bacterial lung infections 6, 20-22. Among these cell types, γδ T cells are more abundant and act an essential role in mucosal immunity in the lung 23, 24. They have been shown to be essential for host defense against *K. pneumoniae*, contributing to bacterial clearance and resistance to infection 25, 26.

The lung is traditionally thought to be sterile, but recent research has revealed that it hosts a diverse microbiota 27, 28. Several studies have shown that the gut microbiota plays a crucial role in maintaining homeostasis and defending against *K. pneumoniae* lung infection through the gut-lung axis, by fermenting dietary components and promoting nutrient absorption, which in turn modulate pulmonary immune responses 29, 30. Furthermore, antibiotic exposure leading to gut dysbiosis has been shown to increase susceptibility to *K. pneumoniae* lung infection 31-33. However, little is known about the interplay between lung microbiota and innate immunity in pulmonary infection. We hypothesize that the lung microbiota acts as a localized and rapid modulator of pulmonary immune response and sought to determine how it influences innate immunity against *K. pneumoniae* in the lung.

In animals, certain metabolites produced by commensal bacteria, particularly SCFAs such as acetate, propionate, and butyrate, regulate epithelial barrier function and modulate both mucosal and systemic immune responses 34, 35. Recent studies have indicated that SCFAs act not only as intracellular modulators via transporters but also as natural ligands for a group of G protein-coupled receptors (GPRs), known as free fatty acid receptors (FFARs), underscoring the intricate link between metabolism and immunity 36, 37. Several studies have shown that gut microbiota transplantation or oral SCFAs administration can enhance host defense against *K. pneumoniae* lung infection 31, 32. Nevertheless, the underlying mechanisms of lung microbiota-derived SCFAs are not yet fully understood.

Here, we show that mice with intratracheal vancomycin-induced lung dysbiosis exhibit increased susceptibility to *K. pneumoniae* and dampened immune responses to *K. pneumoniae*-derived LPS, including decreased IL-17A production and impaired activation of IL-17A^+^ γδ T cells. These effects are attributed to diminished IL-1β secretion from AMs displaying downregulated NF-κB and GPR signaling following lung dysbiosis. Reduced lung microbiota-derived SCFAs caused by lung dysbiosis, along with the predominant expression of FFAR2 in AMs across species, potentially contribute to the underlying mechanism. Furthermore, acetate, the major ligand for FFAR2, enhances IL-1β production in AMs via the FFAR2-PKA-NF-κB axis. Acetate supplementation restores lung dysbiosis-impaired host defense against *K. pneumoniae* in an FFAR2-dependent manner, while mice lacking FFAR2 on AM display compromised resistance to infection. Taken together, our results demonstrate that lung microbiota drives an FFAR2-mediated innate immunity against *K. pneumoniae*, highlighting how lung dysbiosis disrupts this protective network and suggesting therapeutic opportunities via SCFA-FFAR2 targeting.

## Results

### Vancomycin-induced lung dysbiosis impairs host defense against *K. pneumoniae*

**Figure 1:**
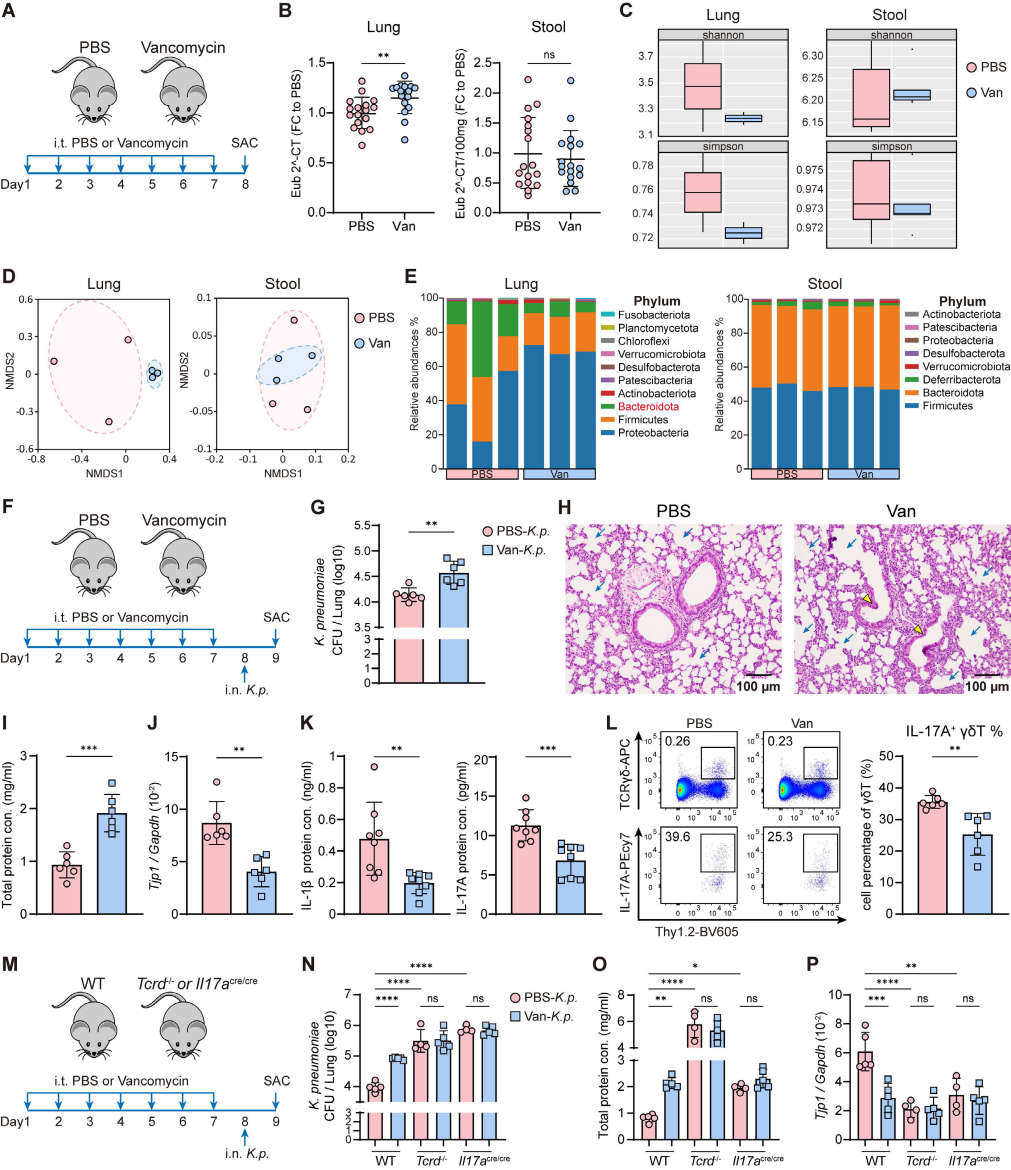
**Intratracheal vancomycin-induced lung dysbiosis impairs host defense against *K. pneumoniae* via γδ T cells and IL-17A.** (**A**) Schematic diagram of protocol involving treatment with intratracheal vancomycin (10 μg/day) treatment for 7 days, and sacrificed one day after the last treatment. (**B**-**E**) The composition of lung and gut microbiota were evaluated by bacteria loads and 16S rRNA sequencing. (**B**) Bacteria loads of the lung (left) and gut (right) microbiota were accessed by qPCR using universal eubacterial (EUB) primers. (**C**) α-diversity of the lung (left) and gut (right) microbiota between the PBS and the Van-treated group as indicated by the Shannon and Simpson indices (n = 3). (**D**) β-diversity of the lung (left) and gut (right) microbiota were shown as Non-metric Multidimensional Scaling (NMDS) (n = 3). (**E**) Relative abundance of the lung (left) and gut (right) microbiota at phylum level between the PBS and Van-treated group (n = 3). (**F**) Illustration of experimental model: Mice were administrated with intratracheal vancomycin treatment for 7 days, followed by intranasal *K. pneumoniae* (5×10^7^ CFU) infection, and sacrificed 24 h later. (**G**-**L**) The ability of host defense and phenotype of inflammatory responses were elevated. (**G**) Pulmonary *K. pneumoniae* bacterial burdens (n = 6). (**H**) H&E staining of lung tissues (bar, 100 μm). Yellow arrow indicates representative bronchial distortion and destruction; blue arrow indicates representative alveolar damage and fusion. (**I**) Total protein in BALF (n = 6). (**J**) Lung *Tjp1* mRNA expression (n = 6). (**K**) Protein levels of IL-1β (left) and IL-17A (right) in BALF (n = 8). (**L**) Representative flow cytometry plots (left) and frequency quantification (right) of lung IL-17A^+^ γδ T cells (Gating from γδ T cells (CD45^+^CD90.2^+^TCRγδ^+^)) (n = 6). (**M**) Experimental design: Intratracheal vancomycin treatment was administrated to C57BL/6 WT mice, γδ T-deficient mice (*Tcrd*^-/-^), or IL-17A-deficient mice (*Il17a*^cre/cre^) for 7 days, followed by intranasal *K. pneumoniae* (5×10^7^ CFU) infection, and sacrificed 24 h later. (**N**-**P**) The ability of host defense was elevated. (**N**) Pulmonary *K. pneumoniae* bacterial burdens (n = 4-6). (**O**) Total protein in BALF (n = 4-6). (**P**) Lung *Tjp1* mRNA expression (n = 4-6). Data are representative of 2-3 independent experiments and values are shown as mean ± SEM; p-value were calculated by unpaired Student's t test (**B**,** G**, **I**-**L**) or one-way ANOVA (**N**-**P**). n.s. Not significant. *p < 0.05, **p < 0.01, ***p < 0.001, ****p < 0.0001.

Vancomycin is one of the most widely prescribed antibiotics in hospitals 38, 39, but its use is associated with drug-resistant nosocomial infections and gut dysbiosis-related diseases 40-43. Despite this, its effects on the lung microbiota and the subsequent impact on host defense against pulmonary infection remain unclear. A study by Hosang *et al*. demonstrated that daily intratracheal antibiotic administration disrupts the lung microbiome without altering the lung-intrinsic cellular immune milieu or gut microbiota 44. Base on this finding, we induced lung dysbiosis by intratracheal administration of vancomycin (Figure 1A). To validate the effect, we extracted bacterial DNA from the lung and stool following treatment and performed real-time PCR and 16S rRNA sequencing. We found higher bacterial loads (Figure 1B) and lower bacterial diversity (Figure 1C) in the lung of the vancomycin-treated group compared to the PBS-treated group, while no differences were detected in the stool. In addition, non-metric multidimensional scaling (NMDS) analysis (Figure 1D) and the profiling of bacterial composition and relative abundance (Figure 1E, Figure S1A) showed that intratracheal vancomycin treatment changed lung microbiota but did not affect the gut microbiota. Our findings demonstrate that intratracheal vancomycin treatment selectively induces lung dysbiosis while leaving the gut microbiota unaffected.

To assess the impact of lung dysbiosis on host defense against *K. pneumonia* lung infection, mice were intranasally challenged with *K. pneumoniae* (5×10^7^ CFU) following vancomycin exposure (Figure 1F). We first confirmed that vancomycin did not have activity against *K. pneumoniae in vitro* (Figure S1B). Further, vancomycin treatment led to increased bacterial burden (Figure 1G, Figure S1C) and exacerbated lung injury following *K. pneumoniae* infection, as evidenced by severe pulmonary edema, the distortion and destruction of bronchial and alveolar walls in the lung airways and parenchyma (Figure 1H), elevated total protein in BALF (Figure 1I), and reduced mRNA expression of tight junction protein 1 (*Tjp1*) in the lung (Figure 1J). Moreover, reduced cytokine production of IL-1β and IL-17A (Figure 1K) were observed, along with a decreased frequency and absolute number of IL-17A^+^ γδ T cells (Figure 1L, Figure S1D) in the lung of vancomycin-treated mice. The susceptibility to *K. pneumoniae* was increased in γδ T cell-deficient (*Tcrd*^-/-^) and IL-17A-deficient (*Il17a*^cre/cre^) mice compared with WT mice (Figure 1M-P, Figure S1E), whereas vancomycin treatment did not further compromise host defense in these knockout strains (Figure 1M-P, Figure S1E). Collectively, these results indicate that vancomycin-induced lung dysbiosis impairs host defense against *K. pneumoniae* infection by disrupting γδ T cell and IL-17A signaling.

### Lung dysbiosis suppresses *K. pneumoniae*-derived LPS-induced immune responses

**Figure 2:**
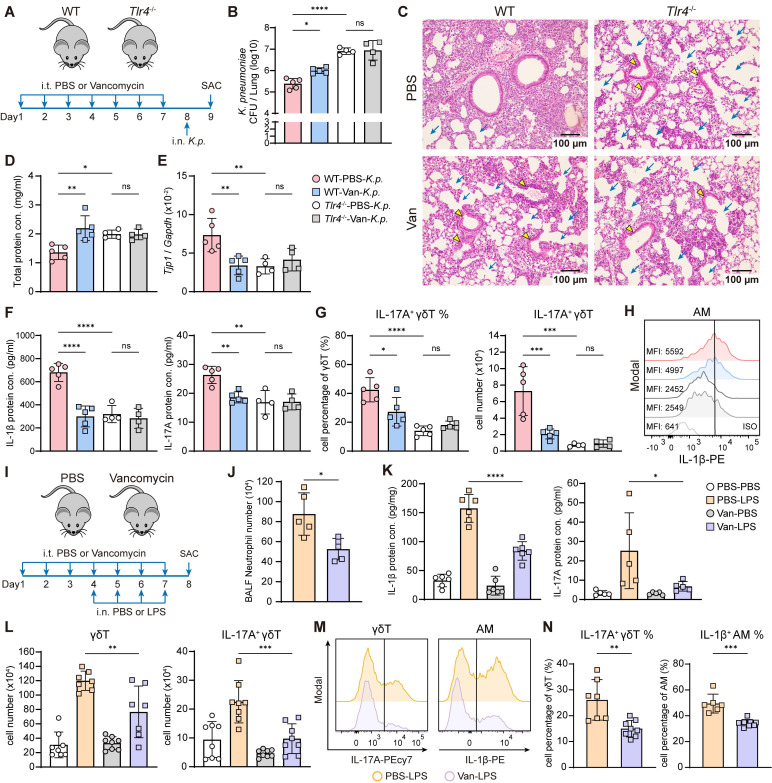
**Lung dysbiosis attenuates LPS-induced immune responses and the activation of IL-1β^+^ AMs and IL-17A^+^ γδ T cells.** (**A**) Experimental design: Intratracheal vancomycin treatment was administrated to C57BL/6 WT mice or TLR4-deficient mice (*Tlr4*^-/-^) for 7 days, followed by intranasal K. pneumoniae (5×10^7^ CFU) infection, and sacrificed 24 h later. (**B**-**H**) The ability of host defense and phenotype of inflammatory responses were elevated. (**B**) Pulmonary *K. pneumoniae* bacterial burdens (n = 4-5). (**C**) H&E staining of lung tissues (bar, 100 μm). Yellow arrow indicates representative bronchial distortion and destruction; blue arrow indicates representative alveolar damage and fusion. (**D**) Total protein in BALF (n = 4-5). (**E**) Lung *Tjp1* mRNA expression (n = 4-5). (**F**) Protein levels of IL-1β (left) and IL-17A (right) in BALF (n = 4-5). (**G**) Frequency (left) and absolute number (right) of lung IL-17A^+^ γδ T cells (n = 4-5). (**H**) Representative histogram of IL-1β expression in AMs (Gating from AMs (CD45^+^Ly6G^-^CD64^+^CD11c^+^SiglecF^+^)) from individual mice. (**I**) Schematic diagram of protocol involving treatment with intratracheal vancomycin (10 μg/day) treatment for 7 days and intranasal LPS (2 μg/day) stimulation in the last 4 days, sacrificed one day after the last treatment. (**J**-**N**) The phenotype of inflammatory responses was elevated. (**J**) BALF neutrophils numbers (n = 5). (**K**) Protein levels of IL-1β (left), IL-17A (right) in lung homogenates or BALF (n = 4-6). (**L**) Absolute number of γδ T cells (left) and IL-17A^+^ γδ T cells (right) in the lung (n = 7-8). (**M**) A Representative histogram of IL-17A expression in γδ T cells (left) and IL-1β expression in AMs (right). (**N**) Frequency quantification of lung IL-17A^+^ γδ T cells (left) and IL-1β^+^ AMs (right) (n = 6-8). Data are representative of 2-3 independent experiments and values are shown as mean ± SEM; p-value were calculated by one-way ANOVA (**B**, **D**-**G**, **K**, **L**) or unpaired Student's t test (**J**, **N**). n.s. Not significant. *p < 0.05, **p < 0.01, ***p < 0.001, ****p < 0.0001.

As a Gram-negative bacterium, LPS of* K. pneumoniae* plays a major role in triggering host innate immune responses through TLR4 signaling 7-9. In TLR4-deficient (*Tlr4*^-/-^) mice (Figure 2A), we observed higher bacterial burden (Figure 2B) and exacerbated lung injury (Figure 2C-E) following *K. pneumoniae* infection, accompanied by reduced protein levels of IL-1β and IL-17A (Figure 2F), as well as a lower frequency and absolute number of IL-17A^+^ γδ T cells in the lung (Figure 2G). Furthermore, IL-1β expression in AMs was also decreased in TLR4-deficient mice (Figure 2H). However, the additional impairment of host defense and immune response caused by vancomycin-induced lung dysbiosis was not evident in the absence of TLR4 (Figure 2B-H). Together, these findings demonstrate that both effective host defense and lung dysbiosis-induced impairment against *K. pneumoniae* rely on LPS-TLR4 signaling.

To further investigate the effect of lung dysbiosis on TLR4 ligand-mediated host defense responses, mice were pretreated intratracheally with vancomycin, followed by intranasal administration of* K. pneumoniae*-derived LPS to induce acute pulmonary inflammation (Figure 2I) 45. The results showed that vancomycin-treated mice exhibited a diminished immune response, characterized by reduced neutrophil infiltration in the BALF (Figure 2J), attenuated mRNA and protein levels of IL-1β, IL-17A and IL-22 in the lung (Figure 2K, Figure S2A-B). Moreover, γδ T cell numbers and both the frequency and absolute number of IL-17A^+^ γδ T cells were all decreased (Figure 2L-N). However, no significant changes were observed in the proportion or number of IL-17A^+^ CD4 T cells following vancomycin exposure (Figure S2C).

We next used *Tcrd*^-/-^ and *Il17a*^cre/cre^ mice to determine whether the vancomycin-induced suppression of LPS-triggered immune responses depends on γδ T cells and IL-17A signaling (Figure S2D). Upon LPS stimulation, we found that γδ T cell- and IL-17A-deficient mice exhibited attenuated immune responses, as evidenced by reduced neutrophil counts in BALF and decreased pulmonary IL-17A mRNA and protein levels compared with WT controls (Figure S2E-J). Nevertheless, no additional immunosuppressive effects of vancomycin treatment were observed in γδ T cell- and IL-17A-deficient mice (Figure S2E-J). These results indicate that LPS-induced acute lung inflammatory response is mediated by γδ T cells and IL-17A signaling, contributing to the vancomycin-induced suppression of immune response in the lung.

The effect of the systemic microbiome on LPS-induce acute inflammatory response was further confirmed using germ-free (GF) mice (Figure S3A). Following LPS stimulation, GF mice also exhibited reduced BALF neutrophil numbers (Figure S3B) and lower mRNA and protein levels of lung IL-1β, IL-17A and IL-22 (Figure S3C-D) compared with specific pathogen-free (SPF) mice. This was accompanied by a reduced frequency and absolute number of γδ T cells and IL-17A^+^ γδ T cells in the lung (Figure S3E-F). However, there were no differences in the proportion or number of IL-17A^+^ CD4 T cells between GF and SPF mice (Figure S3G-H). These observations illustrate that systemic microbiome depletion significantly attenuates the LPS-induced pulmonary innate immune response as well.

Moreover, we also found that lung dysbiosis reduced IL-1β expression in AMs following LPS stimulation, as determined by flow cytometry (Figure 2M-N). These findings indicate that intratracheal vancomycin treatment downregulate LPS-induced acute lung inflammatory response and IL-17A^+^ γδ T cells activation, potentially through the suppression of IL-1β production from AMs 20, 21.

### AMs display restrained NF-κB and GPR signaling associated with IL-17A⁺ γδ T cell suppression

**Figure 3:**
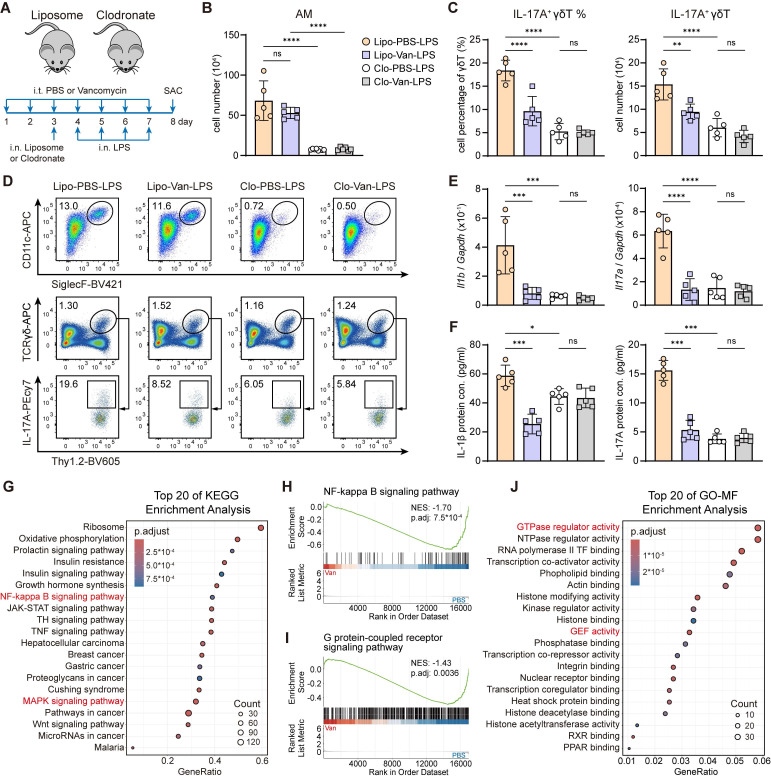
**Suppression of IL-17A^+^ γδ T cell is AM-dependent and linked to dampened NF-κB and GPR signaling.** (**A**) Schematic diagram of protocol involving treatment with intratracheal vancomycin (10 μg/day) treatment for 7 days, following with intranasal clodronate (55 μl) administration to deplete AMs and LPS (2 μg/day) stimulation to induce acute lung inflammation. Mice were sacrificed one day after the last treatment. (**B**-**F**) The phenotype of inflammatory responses was elevated. (**B**) Absolute numbers of AMs in lungs (n = 5). (**C**) Frequency quantification (left) and absolute numbers (right) of lung IL-17A^+^ γδ T cells (n = 5). (**D**) Representative flow cytometry plots of AMs (Gating from CD45^+^Ly6G^-^CD64^+^) and IL-17A^+^ γδ T cells (Gating from γδ T cells (CD45^+^CD90.2^+^TCRγδ^+^)). (**E**) mRNA expression of *Il1b* (left) and *Il17a* (right) in lung homogenates (n = 5). (**F**) Protein levels of IL-1β (left) and IL-17A (right) in BALF (n = 5). (**G**-**J**) Bulk RNA-seq data from AMs isolated from the BALF of PBS- or vancomycin-treated mice following LPS stimulation. (**G**) Top 20 pathways enriched in KEGG enrichment analysis. Barcode plots from GSEA of NF-κB (**H**) and GPR (**I**) signaling pathway. (**J**) Top 20 pathways enriched in GO-Molecular Function enrichment analysis. Data are representative of 2 independent experiments and values are shown as mean ± SEM; p-value were calculated by one-way ANOVA (**B**, **C**, **E**, **F**), the Benjamini-Hochberg method (**G**, **J**) or one-sided Wilcoxon rank-sum test (**H**, **I**). n.s. Not significant. *p < 0.05, **p < 0.01, ***p < 0.001, ****p < 0.0001.

During lung infection, AMs serve as the first line of host defense by detecting, engulfing, and eliminating pathogens. They also produce large amounts of pro-inflammatory cytokines, such as IL-1β, IL-23, which are critical for the activation of IL-17A^+^ γδ T cells and the initiation of downstream immune responses against infection 6, 14-18, 20-22. To determine whether the immunosuppressive effect on IL-17A^+^ γδ T cells activation under lung dysbiosis was mediated by AMs, we administered clodronate intranasally one day before LPS stimulation to deplete AMs following vancomycin treatment (Figure 3A). After AMs depletion (Figure 3B), we observed diminished immune responses, including reduced frequency and number of IL-17A^+^ γδ T cells (Figure 3C-D) and decreased mRNA and protein levels of IL-1β and IL-17A in the lung (Figure 3E-F) compared with liposome controls. However, the vancomycin-induced immunosuppressive effects observed in the liposome controls were absent in the clodronate group (Figure 3C-F). These results indicate that AMs play a crucial role in modulating the LPS-induced inflammatory response and mediating the immune suppression following lung dysbiosis.

To explore the mechanisms underlying the effect of intratracheal vancomycin treatment-induced lung dysbiosis on AMs, we sorted AMs from BALF after intratracheal PBS or vancomycin treatment followed by intranasal LPS stimulation to perform bulk RNA sequencing (Figure S4A). Consistent with findings from previous literature on the impacts of gut microbiota 32, 46, lung dysbiosis exerted a marked effect on the AMs transcriptome (Figure S4B). Kyoto Encyclopedia of Genes and Genomes (KEGG) analysis and gene set enrichment analysis (GSEA) revealed the top enrichment and significant downregulation of NF-κB signaling pathway (Figure 3G-H), MAPK signaling pathway (Figure 3G, Figure S4C) and Th17 cell differentiation (Figure S4D) in AMs from vancomycin-treated mice, in agreement with reduced IL-1β production and IL-17A^+^ γδ T cells activation 47, 48 (Figure 2K-N, Figure S2A). Moreover, Gene Ontology (GO) term enrichment analysis of Molecular Functions (MF) and GSEA showed the top enrichment and significant reduction of G protein-coupled receptor (GPR) signaling pathway (Figure 3I), GTPase regulator activity (Figure 3J, Figure S4E) and guanyl-nucleotide exchange factor (GEF) activity (Figure 3J, Figure S4F) in AMs from vancomycin-treated mice. Together, these observations suggest that lung dysbiosis downregulates the inflammatory signaling in AMs, likely through the suppression of GPR signaling pathway 49, 50.

### Diminished lung microbiota-derived SCFAs following lung dysbiosis and predominant FFAR2 expression in AMs

**Figure 4:**
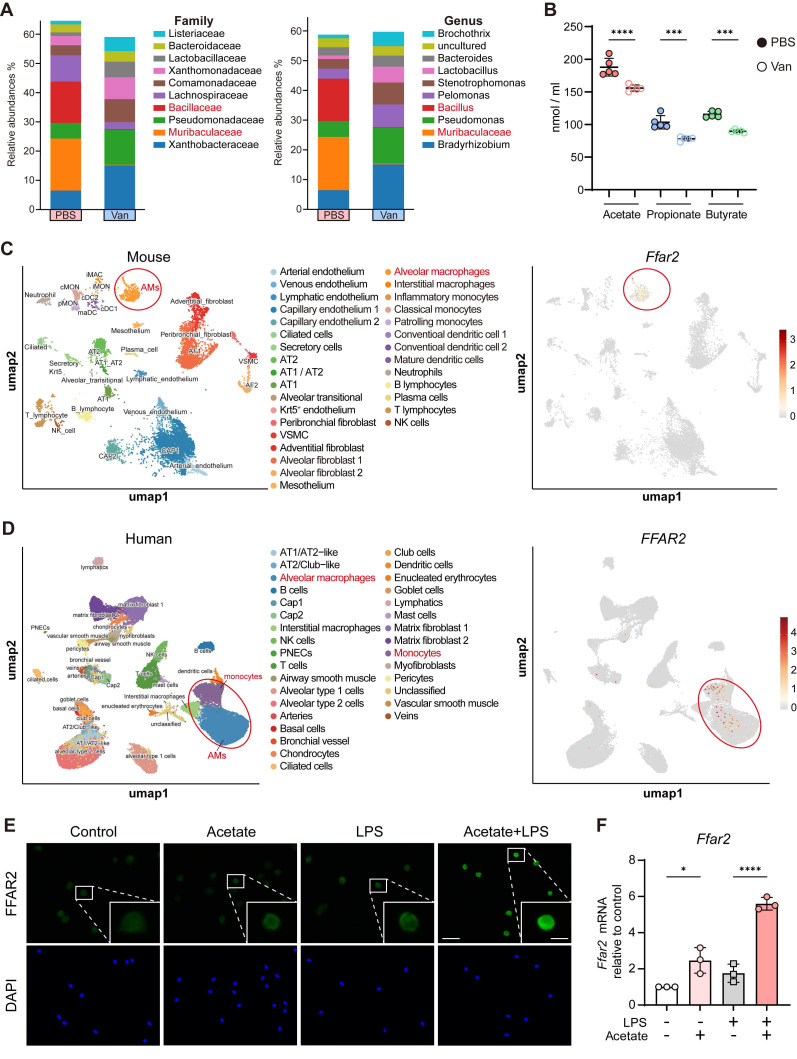
**Diminished lung SCFAs levels following lung dysbiosis and predominant FFAR2 expression in AMs.** (**A**) Grouped relative abundance of the lung microbiota at family (left) and genus (right) levels between PBS and Van-treated group (n = 3). (**B**) Targeted metabolomics analysis of SCFAs in BLAF of PBS and Van-treated mice (n = 5) was analyzed using LC/MS. (**C**) UMAP plot of total WT naïve murine lung cells (left) and feature plot illustrating the enrichment of *Ffar2* (right). The scRNA-seq data was obtained and reanalyzed from GSE262927. (**D**) UMAP plot of total health human lung cells from a broad age healthy donor (left) and feature plot illustrating the enrichment of *FFAR2* (right). The scRNA-seq data was obtained and reanalyzed from GSE161382. (**E**,** F**) Expression of FFAR2 on AMs was evaluated by immunofluorescence (**E**) (Scale bar: 50 μm (left) and 10 μm (right)) and RT-qPCR (**F**) following *ex vivo* treatment with or without acetate (1 mM) for 30 min, followed by LPS (1 μg/ml) for 6 h. Data are representative of 1-2 independent experiments and values are shown as mean ± SEM; p-value were calculated by one-way ANOVA (**B**, **F**). n.s. Not significant. *p < 0.05, **p < 0.01, ***p < 0.001, ****p < 0.0001.

Previous studies have shown that microbiota-derived SCFAs modulate inflammatory response and enhance host defense against pathogen infection 31, 32 through the activation of GPRs, also known as FFARs, including GPR43 (FFAR2) and GPR41 (FFAR3), thereby triggering intracellular signaling pathways 34-37. However, the relationship between lung microbiota-derived SCFAs and FFARs signaling in the lung remains poorly understood. In our study, 16S rRNA sequencing of lung microbiota revealed significant reductions in the genera of *Bacillus* and *Muribaculaceae*, both well-known SCFA-producing bacteria 51, 52, in the vancomycin-treated group (Figure 4A). Furthermore, targeted metabolomics analysis of BALF from PBS- or vancomycin-treated mice using LC-MS demonstrated that the levels of acetate, propionate, and butyrate, the three most abundant SCFAs, were significantly decreased in vancomycin-treated group (Figure 4B).

Moreover, re-analysis of published scRNA-seq datasets from total lung cells of naïve WT mice and healthy humans revealed that FFAR2, a primary receptor for SCFAs, is predominantly expressed in AMs (Figure 4C-D), whereas FFAR3, another SCFA receptor, exhibits minimal to no expression (Figure S5A-B) in both species. Meanwhile, *in vitro* experiments also showed that acetate, the major ligand for FFAR2, upregulated FFAR2 expression in AMs, as confirmed by immunofluorescence staining and RT-qPCR (Figure 4E-F). These findings suggest that the impaired levels of lung microbiota-derived SCFAs due to vancomycin exposure may contribute to the reduced IL-1β production from AM via FFAR2.

### Acetate enhances IL-1β production, inflammasome activation, and NF-κB phosphorylation in AMs via FFAR2

**Figure 5:**
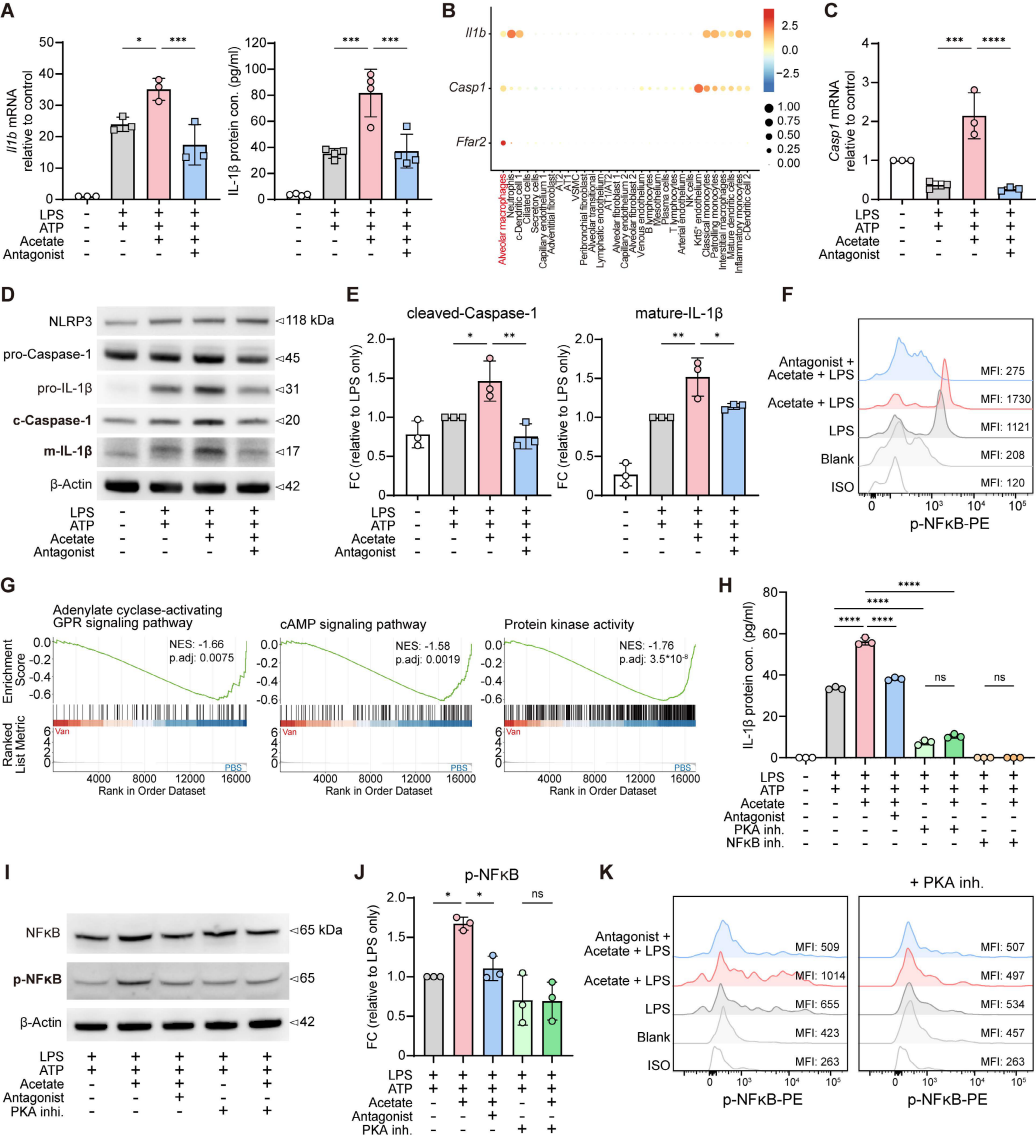
**Acetate enhances IL-1β production in AMs via FFAR2-PKA-NF-κB axis. A**,** C-E** Sorted AMs were pre-incubated with or without FFAR2 antagonist (10 μM) for 30 min, followed by treatment with or without acetate (1 mM) for another 30 min. Subsequently, the cells were stimulated with or without LPS (1 μg/ml) for 6 h (RT-qPCR and Western blot (10^6^ cells/ml)) or 24 h (ELISA (10^5^ cells/ml)), with ATP (2 mM) added in the last 30 min to induce inflammasome activation. The cell lysates and supernatants were then collected for further analysis. (**A**) mRNA expression (left) and protein level (right) of IL-1β in AMs cell lysates or supernatants (n = 3-4). (**B**) Bubble plot of total WT naïve murine lung cells showing the expression levels of* Il1b*, *Casp1* and *Ffar2* across annotated cell types. Bubble size represents the percentage of cells expressing each gene; color intensity indicates mean expression level. The scRNA-seq data was obtained and reanalyzed from GSE262927. (**C**) mRNA expression of *Casp1* in AMs cell lysates (n = 3-4). (**D**,** E**) Immunoblot analysis (**D**) and the quantification of cleaved-caspase-1 (left) and mature-IL-1β (right) in protein extracted from AMs (**E**) (n = 3). (**F**) Phosphorylation of NF-κB (p65) was determined by flow cytometry. Sorted AMs (10^5^ cells/ml) were pre-incubated with or without FFAR2 antagonist (10 μM) for 30 min, followed by treatment with or without acetate (1 mM) for another 30 min. Subsequently, the cells were stimulated with or without LPS (1 μg/ml) for 10 min. (**G**) Barcode plots from GSEA of adenylate cyclase-activating GPR signaling pathway (left)-cAMP signaling pathway (middle)-protein kinase activity (right) in AMs isolated from the BALF of Van-treated versus PBS-treated mice with LPS stimulation. (**H**-**J**) Sorted AMs were pre-incubated for 30 minutes with or without FFAR2 antagonist (10 μM), PKA inhibitor (10 μM) or NF-κB inhibitor (10 μM), followed by treatment with or without acetate (1 mM) for an additional 30 min. Subsequently, the cells were stimulated with or without LPS (1 μg/ml) for 6 h (Western blot (10^6^ cells/ml)) or 24 h (ELISA (10^5^ cells/ml)), with ATP (2 mM) added in the last 30 min to induce inflammasome activation. The cell lysates and supernatants were then collected for further analysis. (**H**) Protein level of IL-1β in AMs supernatants (n = 3). (**I**,** J**) Immunoblot analysis (**I**) and the quantification of NF-κB (p65) phosphorylation in protein extracted from AMs (**J**) (n = 3). (**K**) Phosphorylation of NF-κB (p65) was determined by flow cytometry. Sorted AMs (10^5^ cells/ml) were sequentially treated with or without PKA inhibitor (10 μM), FFAR2 antagonist (10 μM), and acetate (1 mM), each for 30 min, as indicated. Subsequently, the cells were stimulated with or without LPS (1 μg/ml) for 10 min. Data are representative of 1-3 independent experiments and values are shown as mean ± SEM; p-value were calculated by one-way ANOVA (**A**,** C**,** E**,** H**,** J**). n.s. Not significant. *p < 0.05, **p < 0.01, ***p < 0.001, ****p < 0.0001.

To determine how SCFAs regulate IL-1β production from AMs through FFAR2, we focused on acetate, the most abundant SCFA in the lung and the major ligand for FFAR2. AMs were isolated from BALF and subjected to *in vitro* experiments, which showed that acetate treatment increased *Il1b* mRNA expression in AMs (Figure S5C). Under LPS stimulation, acetate further upregulated *Il1b* mRNA expression and enhanced IL-1β protein secretion (Figure 5A). However, GLPG0974, a selective antagonist of FFAR2, effectively attenuated the acetate-enhanced IL-1β production (Figure 5A). Interestingly, acetate exerted the opposite effect in interstitial macrophages (IMs) and bone marrow-derived macrophages (BMDMs), suppressing IL-1β secretion in an FFAR2-independent manner (Figure S5D), highlighting the acetate-FFAR2 axis as a specific regulator of IL-1β production in AMs.

Upon pathogen infection, NLRP3 inflammasome assembly leads to caspase-1 activation, which subsequently cleaves pro-IL-1β into its mature form 53, 54. Re-analysis of published scRNA-seq datasets also revealed that IL-1β, caspase-1, and FFAR2 are highly co-expressed in AMs from both mice (Figure 5B) and humans (Figure S5E), suggesting a conserved regulatory module. Consistently, our RNA-seq analysis showed reduced NOD-like receptor activity in AMs from vancomycin-treated mice (Figure S5F), implying a potential underlying mechanism 53, 54. Furthermore, under LPS stimulation, acetate treatment upregulated* Casp1* mRNA expression in AMs in an FFAR2-dependent manner (Figure 5C). Immunoblot analysis further showed that acetate promoted inflammasome activation, as evidenced by the increased protein levels of cleaved caspase-1 and both pro- and mature IL-1β in AM cell lysates, which were counteracted by the FFAR2 antagonist (Figure 5D-E, Figure S5G). These findings are consistent with the reduced mRNA expression of inflammasome components following vancomycin exposure and AM depletion in the lung (Figure S5H).

We next examined whether acetate regulates NF-κB signaling in AMs, a central transcription factor controlling both pro-IL-1β production and NLRP3 inflammasome activation. NF-κB responds to pattern-recognition receptors (PRRs) and cytokine signals to drive transcription of pro-IL-1β and inflammasome components 53-56. We discovered that acetate treatment further enhanced NF-κB phosphorylation in AMs following LPS stimulation *in vitro*, which was attenuated by the FFAR2 antagonist pretreatment (Figure 5F). These results support the idea that SCFAs modulate the inflammatory responses in AMs through FFAR2.

### PKA-NF-κB signaling mediates acetate-FFAR2 axis-induced IL-1β upregulation in AMs

Activation of FFARs trigger intracellular heterotrimeric G protein signaling cascade through the interactions with multiple downstream effectors. Among them, the adenylate cyclase-cAMP-PKA signaling pathway is the major cascade that modulates inflammatory responses 49, 50, and regulates the function of NF-κB as well 57, 58. GSEA of our RNA-seq data showed this pathway was downregulated in AMs isolated from vancomycin-treated mice (Figure 5G, Figure S5I). To investigate whether acetate-enhanced IL-1β production via FFAR2 in AMs was mediated by PKA-NF-κB signaling pathway, we pretreated H 89 2HCl (PKA inhibitor) and BAY 11-7082 (NF-κB inhibitor) to inhibit the pathway. The results showed that acetate-induced increases in both NF-κB phosphorylation and IL-1β production were attenuated by PKA inhibitor pretreatment, as demonstrated by ELISA of culture supernatants (Figure 5H), immunoblotting of cell lysates (Figure 5I-J), and flow cytometry analysis (Figure 5K). Furthermore, NF-κB inhibitor completely blocked IL-1β production by AMs (Figure 5H).

Besides the natural FFAR2 ligand acetate, the synthetic FFAR2 agonist TUG-1375 also increased IL-1β mRNA expression (Figure S5J) and protein secretion (Figure S5K) from AMs under LPS stimulation. These effects were also attenuated by the pretreatment of PKA inhibitor and totally abolished by NF-κB inhibitor (Figure S5J-K), underscoring the essential role of this pathway. Together, these observations indicate that acetate-induced IL-1β upregulation in AMs, mediated through FFAR2, is driven by the PKA-NF-κB signaling pathway.

### Acetate-FFAR2 axis-increased IL-1β secretion from AMs enhances IL-17A^+^ γδ T cell activation

**Figure 6:**
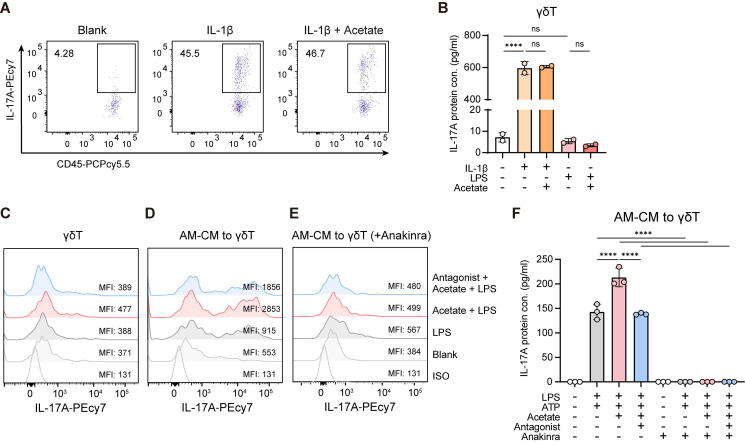
** Acetate promotes IL-17A^+^ γδ T cell activation via FFAR2-mediated increased IL-1β secretion from AMs.** γδ T cells (CD45^+^CD90.2^+^TCRγδ^+^) were sorted from lung lysate of WT C57BL/6 mice. After sorting, γδ T cells (10^5^ cells/ml) were pre-incubated in culture medium supplemented with IL-2 and IL-7 (each 10 ng/ml) for 24 h, followed by various ex vivo experiments. The cells and supernatants were then collected for flow cytometry and ELISA analysis. (**A**,** B**) Subsequently, γδ T cells were stimulated with or without IL-1β (1 ng/ml) or LPS (1 μg/ml) in the presence or absence (30 min pre-treatment) of acetate (1 mM) for 24 h. (**A**) Representative flow cytometry plots of IL-17A^+^ γδ T cells. (**B**) Protein level of IL-17A in γδ T cell supernatants. (**C**) Representative histogram of IL-17A expression in γδ T cells. γδ T cells were pre-incubated with or without FFAR2 antagonist (10 μM) for 30 min, followed by treatment with or without acetate (1 mM) for another 30 min. Subsequently, the cells were stimulated with or without LPS (1 μg/ml) for 24 h. (**D**-**F**) AMs (10^5^ cells/ml) sorted from BALF of WT C57BL/6 mice were pre-incubated with or without FFAR2 antagonist (10 μM) for 30 min, followed by treatment with or without acetate (1 mM) for another 30 min. Subsequently, the cells were stimulated with or without LPS (1 μg/ml) for 24 h, adding ATP (2 mM) in the last 30 min to induce inflammasome activation. Afterward, the AM conditioned medium (AM-CM) was transferred to γδ T cells, which had been pre-treated or not with IL-1 receptor antagonist (Anakinra, 10 μg/ml) for 30 min, and then incubated for additional 24 h. (**D**,** E**) Representative histogram of IL-17A expression in γδ T cells. (**F**) Protein level of IL-17A in γδ T cell supernatants. Data are representative of 2-3 independent experiments and values are shown as mean ± SEM; p-value were calculated by one-way ANOVA (**B**,** F**). n.s. Not significant. *p < 0.05, **p < 0.01, ***p < 0.001, ****p < 0.0001.

To examine whether acetate directly influences the activation of IL-17A^+^ γδ T cells, purified γδ T cells were cultured with acetate in the presence of IL-1β or LPS. Acetate had no effect on IL-17A^+^ cell activation and IL-17A production under either condition (Figure 6A-C). However, γδ T cells exposed to conditioned medium from AMs pretreated with acetate and LPS displayed increased frequencies of IL-17A^+^ cells and elevated IL-17A secretion compared with those cultured with conditioned medium from AMs treated with LPS alone (Figure 6D, F). This enhancement was lost when AMs were preincubated with the FFAR2 antagonist (Figure 6D, F). Moreover, blocking IL-1β signaling in γδ T cells with the IL-1 receptor antagonist anakinra completely abolished IL-17A^+^ cell activation and IL-17A production (Figure 6E-F). Collectively, these results demonstrate that acetate indirectly promotes IL-17A^+^ γδ T cell activation via FFAR2-mediated increased IL-1β secretion from AMs.

### Acetate supplementation restores lung dysbiosis-impaired immune responses and host defense via FFAR2

**Figure 7:**
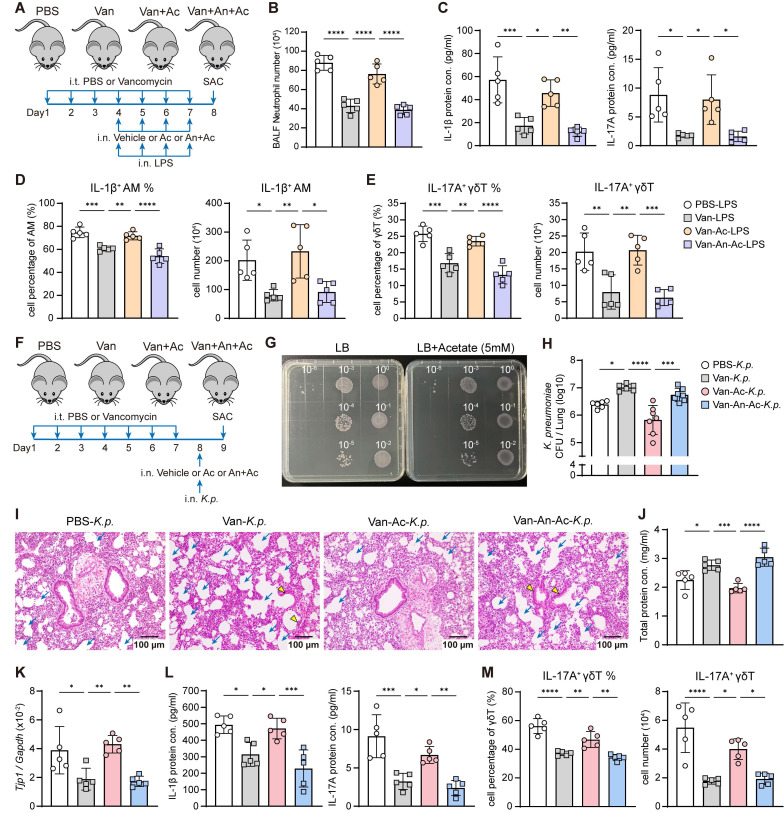
**Acetate supplementation recovers lung dysbiosis-suppressed immune responses and host defense via FFAR2.** (**A**) Schematic diagram of protocol outlining intratracheal vancomycin (10 μg/day) treatment for 7 days, followed by intranasal administration of FFAR2 antagonist (An, 100 μM/55 μl), acetate (A, 5 mM/55 μl) supplementation, and LPS (2 μg/day) stimulation to induce acute lung inflammation in the last 4 days, with sacrifice occurring one day after the final treatment. (**B**-**E**) The phenotype of inflammatory responses was elevated. (**B**) BALF neutrophils numbers (n = 5). (**C**) Protein levels of IL-1β (left) and IL-17A (right) in BALF (n = 5). (**D**, **E**) Frequency quantification (left) and absolute numbers (right) of lung IL-1β^+^ AMs (**D**) and IL-17A^+^ γδ T cells (**E**) (n = 5). (**F**) Schematic diagram of protocol involving intratracheal vancomycin (10 μg/day) treatment for 7 days, followed by intranasal administration of FFAR2 antagonist (An, 100 μM/55 μl) and acetate (Ac, 5 mM/55 μl) supplementation one day after the final antibiotic treatment. Subsequently, after 3 h, intranasally infected with *K. pneumoniae* (5×10^7^ CFU) for 24 h. (**G**) *K. pneumoniae* (2×10^5^ CFU/ml) was incubated in LB broth with or without acetate (5 mM) for 16 h. Subsequently, the cultures were centrifuged, resuspended in 500 μl PBS, and subjected to serial dilution. Then, 10 μl of each indicated dilution was inoculated on the agar plate and incubated for another 16 h. (**H**-**M**) The ability of host defense and phenotype of inflammatory responses were elevated. (**H**) Pulmonary *K. pneumoniae* bacterial burdens (n = 6-7). (**I**) H&E staining of lung tissues (bar, 100 μm). Yellow arrow indicates representative bronchial distortion and destruction; blue arrow indicates representative alveolar damage and fusion. (**J**) Total protein in BALF (n = 5). (**K**) Lung *Tjp1* mRNA expression (n = 5). (**L**) Protein levels of IL-1β (left) and IL-17A (right) in BALF (n = 5). (**M**) Frequency (left) and absolute number (right) of lung IL-17A^+^ γδ T cells (n = 5). Data are representative of 2 independent experiments and values are shown as mean ± SEM; p-value were calculated by one-way ANOVA (**B**-**E**, **H**, **J**-**M**). n.s. Not significant. *p < 0.05, **p < 0.01, ***p < 0.001, ****p < 0.0001.

Given that lung dysbiosis impairs immune response and host defense by disrupting AM-γδ T cell activation, potentially through reduced SCFAs availability and attenuated FFAR2 signaling, we next examined whether acetate supplementation could restore the lung dysbiosis-induced immunosuppressive effects *in vivo*. Intranasal acetate supplementation reversed the vancomycin-induced suppression of pulmonary immune responses to LPS (Figure 7A). This restoration was evidenced by increased BALF neutrophil numbers (Figure 7B), elevated BALF protein levels of IL-1β and IL-17A (Figure 7C), and higher frequency and absolute number of IL-1β^+^ AMs (Figure 7D) and IL-17A^+^ γδ T cells (Figure 7E) in the lung. However, the restoration of immune responses by acetate was abrogated by intranasal FFAR2 antagonist pretreatment (Figure 7B-E).

To further assess the role of acetate in host defense against *K. pneumoniae* infection under lung dysbiosis, *in vivo* acetate supplementation was performed (Figure 7F). We confirmed that acetate did not affect the viability of *K. pneumoniae in vitro* (Figure 7G). However, intranasal acetate supplementation effectively counteracted the vancomycin-induced impairment of host defense against *K. pneumoniae* infection, as indicated by a reduced lung bacterial burden (Figure 7H), ameliorated lung damage (Figure 7I-K), increased IL-1β and IL-17A cytokine levels (Figure 7L), and elevated IL-17A^+^ γδ T cells in the lung (Figure 7M). These protective effects were abolished by intranasal FFAR2 antagonist pretreatment (Figure 7H-M), confirming that intranasal acetate supplementation can reverse vancomycin-induced impairment of immune response and host defense via FFAR2 *in vivo*.

**Figure 8:**
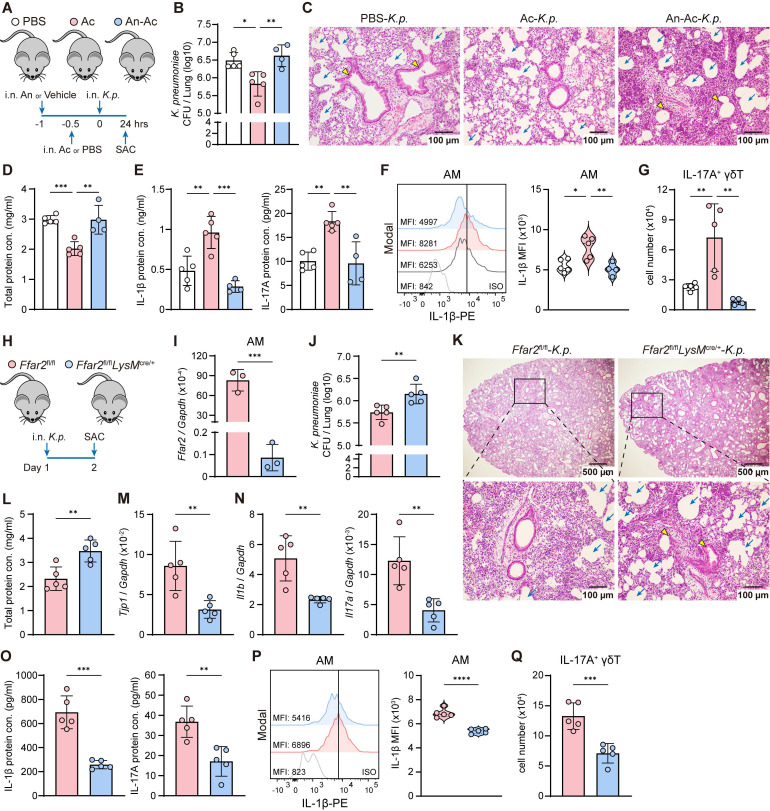
** AM acetate-FFAR2 axis sustains host defense against *K. pneumoniae*.** (**A**) Schematic diagram of protocol involving intranasal administration of FFAR2 antagonist (An, 100 μM/55 μl) and acetate (Ac, 5 mM/55 μl) supplementation following with intranasally infected with *K. pneumoniae* (5×10^7^ CFU) for 24 h. (**B**-**G**) The ability of host defense and phenotype of inflammatory responses were elevated. (**B**) Pulmonary *K. pneumoniae* bacterial burdens (n = 4-5). (**C**) H&E staining of lung tissues (bar, 100 μm). Yellow arrow indicates representative bronchial distortion and destruction; blue arrow indicates representative alveolar damage and fusion. (**D**) Total protein in BALF (n = 4-5). (**E**) Protein levels of IL-1β (left) and IL-17A (right) in BALF (n = 4-5). (**F**) Representative histogram (left) and quantification (right) of IL-1β expression in AMs (n = 4-5). (**G**) Absolute number of lung IL-17A^+^ γδ T cells (n = 4-5). (**H**) Experimental design: *Ffar2*^fl/fl^ and *Ffar2*^fl/fl^*LysM*^cre/+^ mice were intranasally infected with *K. pneumoniae* (5×10^7^ CFU) and sacrificed 24 h later. (**I**) mRNA expression of *Ffar2* in AMs sorted from *Ffar2*^fl/fl^ or *Ffar2*^fl/fl^*LysM*^cre/+^ mice (n = 3). (**J**-**Q**) The ability of host defense and phenotype of inflammatory responses were elevated. (**J**) Pulmonary *K. pneumoniae* bacterial burdens (n = 5). (**K**) H&E staining of lung tissues (bar, 500 μm (above), 100 μm (below)). Yellow arrow indicates representative bronchial distortion and destruction; blue arrow indicates representative alveolar damage and fusion. (**L**) Total protein in BALF (n = 5). (**M**) Lung *Tjp1* mRNA expression (n = 5). (**N**, **O**) mRNA expression (**N**) and protein levels (**O**) of IL-1β (left) and IL-17A (right) in lung lysates or BALF (n = 5). (**P**) Representative histogram (left) and quantification (right) of IL-1β expression in AMs (n = 5). (**Q**) Absolute number of lung IL-17A^+^ γδ T cells (n = 5). Data are representative of 2 independent experiments and values are shown as mean ± SEM; p-value were calculated by one-way ANOVA (**B**, **D**-**G**) or unpaired Student's t test (**I**, **J**, **L**-**Q**). n.s. Not significant. *p < 0.05, **p < 0.01, ***p < 0.001, ****p < 0.0001.

To evaluate whether acetate enhanced host defense against *K. pneumoniae* infection under lung microbiome homeostasis, we administered FFAR2 antagonist and acetate intranasally without vancomycin treatment (Figure 8A). Acetate treated mice exhibited lower lung bacterial burden (Figure 8B), attenuated lung injury (Figure 8C-D), increased IL-1β and IL-17A protein levels (Figure 8E), and elevated IL-1β expression in AMs (Figure 8F) along with greater numbers of IL-17A^+^ γδ T cells (Figure 8G) in the lung compared with control mice. All of these effects were abolished by intranasal FFAR2 antagonist pretreatment (Figure 8B-G). These observations demonstrate that acetate enhances host defense against *K. pneumoniae* infection by modulating immune cell activation and cytokine production through FFAR2.

### FFAR2-deficient in AMs increases susceptibility to *K. pneumoniae*

Next, we examined the role of FFAR2 in AMs during *K. pneumoniae* lung infection *in vivo* using *Ffar2*^fl/fl^*LysM*^cre/+^ mice, with *Ffar2*^fl/fl^ mice as controls (Figure 8H). FFAR2 expression was markedly downregulated in AMs from *Ffar2*^fl/fl^*LysM*^cre/+^ mice compared with *Ffar2*^fl/fl^ controls (Figure 8I). These *Ffar2*^fl/fl^*LysM*^cre/+^ mice exhibited compromised resistance to *K. pneumoniae* infection, as indicated by increased lung bacterial burden (Figure 8J), more severe lung damage (Figure 8K), elevated BALF total protein level (Figure 8L), and reduced pulmonary *Tjp1* expression (Figure 8M). These mice also showed decreased IL-1β and IL-17A mRNA (Figure 8N) and protein levels (Figure 8O), diminished IL-1β expression in AMs (Figure 8P), and fewer IL-17A^+^ γδ T cells in the lung (Figure 8Q). Together, these results demonstrate that FFAR2 expression in AMs is essential for maintaining antibacterial immunity and effective host defense against *K. pneumoniae* infection.

## Discussion

**Figure 9:**
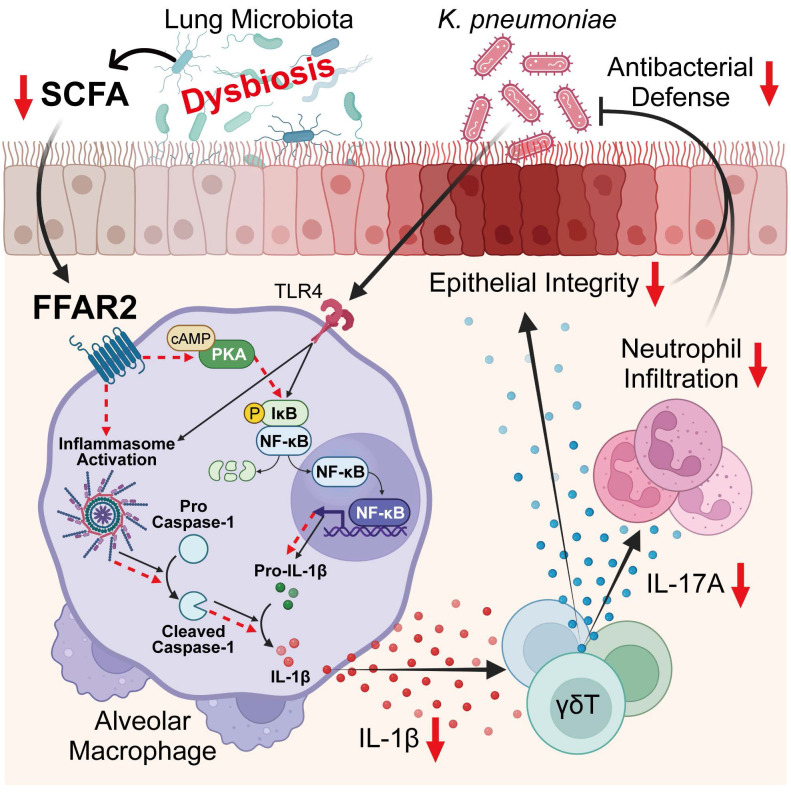
**Lung microbiota coordinates AM and γδ T cell activation against *K. pneumoniae* via SCFA-FFAR2 axis.** Regulatory mechanism of SCFA-FFAR2 signaling in AMs. Lung microbiota-derived SCFAs, particularly acetate, orchestrate AM and γδ T cell activation to enhance host defense against *K. pneumoniae* lung infection. Acetate engages FFAR2 on AMs to activate the PKA-NF-κB signaling pathway, enhancing NF-κB phosphorylation, pro-IL-1β production, and inflammasome activation, which together increase IL-1β secretion and amplify downstream immune responses. This cascade subsequently promotes IL-17A production from γδ T cells, thereby driving neutrophil-mediated defense and preserving epithelial barrier integrity to protect against infection. In contrast, vancomycin-induced lung dysbiosis lowers pulmonary SCFA levels, thereby dampening this immune network and impairing resistance to *K. pneumonia*e infection.

In this study, we employed unbiased approaches by integrating 16S rRNA-seq, bulk RNA-seq, reanalysis of published scRNA-seq datasets, and targeted metabolomics profiling to objectively dissect lung microbiota-immune interactions. We reveal a crucial role of the lung microbiota in orchestrating innate immune defense against *K. pneumoniae*, identifying a lung microbiota-derived SCFA-FFAR2 axis that locally coordinates AM activation and γδ T cell-driven IL-17A responses to sustain antibacterial immunity and mucosal barrier integrity. Mechanistically, the SCFA acetate engages FFAR2 on AMs to activate the PKA-NF-κB signaling cascade, promoting pro-IL-1β transcription, inflammasome activation, and IL-1β secretion. This macrophage-derived IL-1β, in turn, drives IL-17A production from γδ T cells, facilitating neutrophil recruitment and epithelial integrity to protect against lung infection. Conversely, vancomycin-induced lung dysbiosis reduces local SCFA levels, thereby disrupting this immune circuit, leading to impaired AM-γδ T cell activation and compromised host defense against *K. pneumoniae* (Figure 9). Together, these findings uncover a local metabolic-immune network in the lung that mirrors the well-established gut-microbiota-immune axis, highlighting FFAR2-dependent SCFA signaling as a potential therapeutic target for restoring host protection after antibiotic perturbation.

Over the past decades, gut microbiota has been recognized as a crucial regulator in various diseases and immune modulation 59. Through the gut-lung axis, it can modulate lung immune response against *K. pneumoniae* infection 29-33. However, little is known about the role of lung microbiota in respiratory infection. Although several studies have reported a correlation between lung microbiota and pulmonary infectious disease 27, 28, 60, 61, implicating that lung dysbiosis caused by disease or antibiotic exposure increases susceptibility to pathogen infection, the underlying mechanisms remain poorly understood. Our work demonstrates that lung microbiota itself, independent of gut influence, directly shapes innate immunity via SCFA-FFAR2 axis during *K. pneumoniae* lung infection.

Microbiota-derived SCFAs exert broad immunomodulatory functions to shape the immune response. They play a beneficial role in maintaining immune homeostasis through FFARs signaling or intracellular modifications 34, 35. Previous studies have showed that SCFAs enhance the phagocytosis ability of macrophages via FFAR2, thereby promoting protection against *K. pneumoniae* infection 31, 32, 62. We expand this paradigm by demonstrating that SCFAs regulate IL-1β production from AMs through FFAR2, triggering the subsequent immune responses to defend against *K. pneumoniae* lung infection.

Besides FFAR signaling, SCFAs can also enter cells through passive diffusion or via transmembrane carriers and lactate transporters to directly bind to intracellular histone deacetylases (HDACs) and manipulate their activity 34-36. By mediating histone acetylation and regulating chromatin accessibility in different cells, SCFAs influence gene expression and various cellular functions, including cell differentiation, inflammation, antimicrobial responses, fatty acid metabolism, oxidative stress and other related processes 63-65. Previous works have already shown that SCFAs can downregulate pro-inflammatory response by HDACs inhibition in liver and intestinal macrophages 66, 67. In our study, GO-term enrichment analysis revealed top-enriched pathways related to histone-modifying activity and HDACs binding between AMs from PBS-treated and Van-treated mice (Figure 3 J). Although the potential contribution of SCFAs to HDAC regulation in AMs cannot be ruled out, our data suggest that FFAR2-dependent and HDAC-mediated pathways may cooperate to fine-tune the inflammatory plasticity of AMs.

Previous studies have suggested that SCFAs exert anti-inflammatory effects by inhibiting stimulus-induced cytokine and chemokine production and by suppressing immune cell activation and recruitment 34, 35. Consistent with prior reports, we observed reduced IL-1β secretion from IMs and BMDMs following acetate treatment 67-69 independent of FFAR2. In contrast, acetate-FFAR2 signaling seems to promote the pro-inflammatory cytokine IL-1β production from AMs, thereby enhancing anti-microbial immune response. AMs are the resident immune cells in the lung and serve as the first line of defense against invading pathogens. Upon bacterial infection, AMs respond rapidly by engaging in phagocytosis and producing pro-inflammatory cytokines, which are responsible for innate lymphocytes activation and neutrophils recruitment. These boost pathogen clearance and support the maintenance of lung epithelial integrity 14-19. In light of our findings, we propose that the pro-inflammatory effects of SCFAs-FFAR2 signaling in AMs represent a protective mechanism that reinforces host defense during the early phase of infection. According to previous studies, following acute inflammation, resident AMs are largely lost and replaced by IMs and monocyte-derived macrophages, which exhibit distinct functional profiles but collectively contribute to restore lung homeostasis during the resolution phase 17-19. Taken together, our findings implicate that SCFAs may play a crucial role in modulating pulmonary inflammatory response through immune cell dynamics.

Activation of the NLRP3 inflammasome is essential for IL-1β maturation and secretion 53, 54. Although our data demonstrate that acetate-FFAR2 signaling enhances inflammasome activation, the precise mechanism warrants further investigation. It is conceivable that this pathway might modulate damage-associated molecular patterns (DAMPs), such as K^+^ efflux or Ca^2+^ influx, which are both known NLRP3 inflammasome triggers 53, 54. While inflammasome activation was mainly detected in AMs in our study, we do not exclude that the neutrophils also contribute to caspase-1-mediated IL-1β production 62, 70. However, depletion of AMs confirms their central role in mediating inflammatory responses during the early stage of *K. pneumoniae* lung infection. Reduced IL-22 levels in vancomycin-treated and GF mice (Figure S2B, Figure S3C, D), and previous studies showing that acetate can enhance caspase-1-mediated IL-18 production 71 further suggest that SCFA signaling integrates multiple cytokine axes, including IL-18 and IL-22, to promote epithelial repairment.

Intratracheal vancomycin treatment markedly decreased the lung microbiota abundance of *Bacillus* and *Muribaculaceae* (Figure 4A), two well-recognized SCFA-producing taxa 51, 52, accompanied by significant reduction in acetate, propionate, and butyrate levels in the lung (Figure 4B). Although these findings implicate the correlation between vancomycin-induced compositional shift of microbiota and impaired SCFA levels, we did not identify or functionally validate which bacterial species are directly responsible for the observed metabolite changes. Therefore, future studies should isolate and characterize the specific lung commensals capable of SCFA biosynthesis and determine their causal role in shaping AM responses through FFAR2 signaling.

Re-analysis of published scRNA-seq datasets revealed that FFAR2 is also predominantly expressed in human AMs (Figure 4D). Moreover, IL-1β, caspase-1, and FFAR2 were highly co-expressed in AMs from both mice and humans (Figure 5B, Figure S5E), suggesting a conserved signaling module across species. Previous studies have reported that SCFAs suppress IL-1β production in THP-1 and human monocytes 72, 73, yet the role of SCFA-FFAR2 signaling in human AMs has not been explored. These observations raise the possibility that human AMs may perform similar physiological functions as their murine counterparts, underscoring translational relevance and warranting further investigation.

In conclusion, lung microbiota emerges as a direct modulator of pulmonary innate immunity to enhance antibacterial defense against *K. pneumoniae*. Lung dysbiosis disrupts this protective metabolic-immune network, leading to impaired host defense. These insights reveal a novel lung-intrinsic mechanism of microbial-immune communication and underscore the therapeutic potential of SCFA supplementation or FFAR2 targeting to restore mucosal defense after antibiotic perturbation.

## Materials and Methods

### Mice

7 to 8 weeks-old WT SPF and germ free (GF) C57BL/6 mice were purchased from National Laboratory Animal Center (Taipei, Taiwan). *Tcrd*^-/-^ and *Tlr4*^-/-^ mice were purchased from Jackson Laboratory. *Il17*^cre/+^ and *LysM*^cre/+^ mice were kindly provided by Dr. Jr-We Shui (Institute of Biomedical Sciences, Academia Sinica, Taipei, Taiwan). To generate IL-17-deficient mice (*Il17*^cre/cre^), heterozygous *Il17*^cre/+^ mice were intercrossed to produce homozygous *Il17*^cre/cre^ mice, which represent the knock-in/knock-out condition. *Ffar2*^fl/fl^ mice were generated from the Transgenic Core Facility of the Institute of Molecular Biology in Academia Sinica (Taipei, Taiwan). All mouse strains were backcrossed to a C57BL/6 background; except for GF mice, all others were maintained under specific pathogen-free conditions in the animal facility at Academia Sinica. Both male and female mice were used in all experimental groups. For genotype comparisons, conditional knockout mice were analyzed against flox/flox Cre^-^ littermate controls. Whole-body knockout mice were bred separately but co-housed with wild-type mice in the same facility room from weaning until the end of the experiment to minimize environmental and microbiome variation. All mouse experiments were approved by Academia Sinica Institutional Animal Care and Use Committee (IACUC, 20-11-1549, 24-10-2312), and all experiments were performed according to the guidelines of IACUC. Mouse strains used are listed in Table S1.

### Intratracheal vancomycin treatment

Intratracheal (i.t.) vancomycin (10 μg/55 μl PBS once daily, Sigma-Aldrich) treatment per day for 1 week to disturb lung commensal microflora as described 44. Control groups received sterile PBS. After treatment, lung and fecal samples were collected to confirm the efficiency of microbiota disturbance using qPCR and 16S rRNA sequencing. BALF samples were collected to assess the concentration of microbiota-derived SCFAs in the lung.

### *K. pneumoniae* infection and LPS stimulation

*Klebsiella pneumoniae* (ATCC 13883) was cultured in Luria broth (LB) at 37 °C with an agitation speed of 200 rpm under aerobic conditions. After overnight culture, the bacteria were washed with sterile PBS and adjusted for the infection dose (5×10^7^ CFU/mouse) on the optical density at OD600nm and administered intranasally into anesthetized mice with 55 µl PBS. Control groups received sterile PBS. For LPS model, intranasal (i.n.) administration of LPS from *K. pneumoniae* (2 μg /55 μl PBS once daily, Sigma-Aldrich) for 4 consecutive days to induce acute lung inflammation as described 45. Control groups received sterile PBS.

After 24 h post infection, mice were euthanized by an intraperitoneal injection (i.p.) of ketamine and xylazine. BALF was collected using 500 μl PBS with protease inhibitor for cytokine measurements and neutrophil cell counting. Whole lungs were then harvested, part of which was used for histology, and the remainder was homogenized for bacterial burden determination, flow cytometry, and RNA extraction.

### Models of AMs depletion

WT mice were pretreated with intratracheal vancomycin or PBS, followed by intranasal administration of 55 μl liposomal clodronate (Encapsula Nanosciences) (Table S1) to deplete alveolar macrophages in the lung, or control liposomes as control. The cell number of alveolar macrophages in lung were analyzed by flow cytometry.

### Bacterial DNA extraction and qPCR quantification

Whole Lung sample was harvested and homogenized in 800 μl PBS with 500 μl of 1 mm zirconia/silica beads (BioSpec) in 2 ml polypropylene microcentrifuge tube (BioSpec) using Mini-Beadbeater-24 (BioSpec) for 20 s, twice. The homogenate was centrifuged at 500 g for 5 min to pellet the tissue, and the supernatant was transferred to a new 1.5 ml microcentrifuge tube and centrifuge at 500 g for 5 min again to pellet the cells. The resulting supernatant was then transferred to a new 2 ml tube and centrifuged at 8000 g for 10 min to pellet bacteria. The supernatant was removed, and the bacterial DNA was extracted from the pellet using the QIAamp Fast DNA Stool Kit (QIAGEN, Denmark) according to the manufacturer's instructions. Fecal samples (50-100 mg) were used for extraction of gut microbial genomic DNA using the QIAamp Fast DNA Stool Kit (QIAGEN, Denmark) with the manufacturer's instructions.

Both lung and stool bacterial 16S rRNA genes of the V3-V4 region were amplified using the KAPA HiFi HotStart PCR Kit (Roche) according to the manufacturer's instructions, with Eubacteria primers 340F and 514R as templates (Table S2) 74. The following cycler program was used: initial denaturation at 95 °C for 3 min, 25 cycles of denaturation at 95 °C for 30 s, annealing at 57 °C for 30 s and extension at 72 °C for 30 s, followed by a final extension at 72 °C for 5 min.

After amplified, qPCR assay was performed on an TOptical real-time PCR system (Biometra). Reaction mixtures contained 7.5 μl SYBR Green qPCR Master Mix (LabStar), 0.75 μl of each 5 μM 340F and 514R Eubacteria primers, 1 μl H_2_O, and 5 μl of amplified DNA. The cycling conditions included initial denaturation of 3 min at 95 °C followed by 40 cycles of denaturation at 96 °C for 5 s, annealing at 60 °C for 20 s, and extension at 72 °C for 20 s. We evaluated the bacterial quantity by comparing the Ct values of the Van-treated to the PBS group. Reagents and materials used are listed in Table S1.

### 16S rRNA amplicon generation and sequencing

To generate 16S rRNA amplicon, two amplified PCR were performed. The 16S rRNA gene of V3-V4 region was amplified using specific primers (341F and 805R) (Table S2) with an overhang linker sequence for library preparation. Two amplicon PCR reactions were carried out in 25 μl reactions with 12.5μl of KAPA HiFi HotStart Ready Mix (Roche), 5 μM of forward and reverse primers, and about 20 ng DNA template. The amplification was performed with 95 °C for 3 min, and 25 cycles of denaturation at 95 °C for 30 s, annealing at 55 °C for 30 s, and elongation at 72 °C for 30 s, followed by 5 min extension at 72 °C. The 1st stage amplicons were purified by AMPure XP system (Beckman) and followed by Nextera XT Index Kit v2 Set (Illumina, San Diego, CA, USA), and the quality of the libraries was assessed on a Qubit 2.0 Fluorometer (Thermo Scientific, Waltham, MA, USA) and Qsep400 with N1 High Sensitivity Cartridge Kit (Bioptic Inc., Taiwan). The qualified libraries were then sequenced on an Illumina MiSeq platform with the 300 bp paired-end reads generated by Genomics, BioSci & Tech Co., New Taipei City, Taiwan.

### SCFAs metabolomic analysis

BALF samples were collected by flushing the airways 3 times with 1 ml of sterile PBS (37 °C) using a 1 ml syringe from PBS or Van-treated mice. Further, the samples were derivatized with 3-nitrophenylhydrazine (3NPH) and analyzed by LC-MS/MS system. The LC system used for analysis was ultra-performance liquid chromatography (UPLC) system (ACQUITY UPLC, Waters, Millford, MA). The sample was separated with ACQUITY UPLC BEH C18 column (particle size 1.7 μm, 2.1 × 100 mm, Waters). The UPLC system was coupled online to the Waters Xevo TQ-XS triple quadrupole mass spectrometer (Waters, Milford, USA) with Unispray TM ionization source. Acetic acid-d3, butyric acid-d7 and Propionic acid-d5 were used as internal standards. Characteristic MS transitions were monitored using negative multiple reaction monitoring (MRM) mode for 3NPH derivatized short-chain fatty acids (SCFAs) acetic acid (m/z, 194 > 137), Propionic acid (208 > 137), butyric acid (222 > 137), Isobutyric acid (222 > 137), 2-Methylbutyric acid (236 > 137), Valeric acid (236 > 137), Isovaleric acid (236 > 137), 3-Methylvaleric acid (250 > 137), Hexanoic acid (250 > 137), Isohexanoic acid (250 > 137), 13C6-Acetic acid (200 > 143), 13C6-Propionic acid (214 > 143), 13C6-Butyric acid (228 > 143), 13C6-Isobutyric acid (228 > 143), 13C6-2-Methylbutyric acid (242 > 143), 13C6-Valeric acid (242 > 143), 13C6-Isovaleric acid (242 > 143), 13C6-3-Methylvaleric acid (256 > 143), 13C6-Hexanoic acid (256 > 143), and 13C6-Isohexanoic acid (256 > 143).Data acquisition and processing were performed using MassLynx version 4.2 and TargetLynx software (Waters Corp.).

### Lung *K. pneumoniae* bacterial burden

Left upper lobe of lungs were harvest and homogenized in 200 μl PBS with 100 μl of 1 mm zirconia/silica beads (BioSpec) in 2 ml polypropylene microcentrifuge tube using Mini-Beadbeater-24 (BioSpec) for 20 s, twice. The homogenate was centrifuged at 500 g for 5 min to pellet the tissue, and the supernatant was transferred to a new 1.5 ml microcentrifuge tube and centrifuge at 500 g for 5 min again to pellet the cells. The resulting supernatant was processed to quantify bacterial burdens by inoculating serial dilutions of lung homogenates onto Klebsiella ChromoSelect Selective Agar plate (Merck) supplemented with Carbenicillin (50 μg/ml) (Table S1). Colonies were counted after overnight incubation at 37 °C.

### Flow cytometry

Homogenized lungs, prepared by cutting with a surgical blade, were incubated with DNase/Collagenase at 37 °C for 45 min, followed by filtration through a 70 µM cell strainer and red blood cell lysis to obtain a single-cell suspension. For measuring intracellular IL-17A and IL-1β expression, cells were then stimulated with 100 ng/ml PMA (Sigma-Aldrich), 1 µg/ml ionomycin (Sigma-Aldrich) and 0.67 µl/ml GolgiStop (BD) in 10% FBS complete RPMI-1640 medium for 3 h. Further, cells were then stained with Fixable Viability Dye eFluorTM 780 (1:1000 dilution, Thermo Fisher Scientific) and various combinations of the following anti-mouse antibodies for surface marker: CD45, Thy1.2, TCR γδ, Ly6G, CD64, F4/80, CD11c, SiglecF. After 30 min of surface staining, the cells were fixed and permeabilized using an Intracellular Fixation and Permeabilization Kit (Invitrogen) for intracellular staining, followed by staining with antibodies against IL-17A and IL-1β. For analysis of p-NF-κB, AMs were fixed by 4% paraformaldehyde and permeabilized by methanol followed by staining with antibodies against p-NF-κB. After staining, cells were washed, resuspended in FACs buffer, and acquired using LSR II flow cytometer (BD Biosciences). Data were analyzed with Flowjo V10.4. Antibodies and reagents used are listed in Table S1.

### FACS sorting

Cell suspensions of lung or BALF were further purify the cells by removing the debris using Percoll^TM^ density gradient media (Cytiva). Then were stained with various combinations of the following anti-mouse antibodies for surface marker: γδ T cells (CD45^+^Thy1.2^+^TCRγδ^+^), AMs (CD45^+^Ly6G^-^CD64^+^F4/80^+^SiglecF^+^), Interstitial Macrophages (IMs) (CD45^+^Ly6G^-^CD64^+^ F4/80^+^SiglecF^-^). After 30 min of staining, followed by washing and sorting of the cells using a FACSAria^TM^ III Cell Sorter (Becton Dickinson). Sorted γδ T cells and IMs from lung lysates were used for *in vitro* experiment. RNA was extracted from FACS-sorted AMs from BALF for bulk RNA-sequencing. Antibodies and reagents used are listed in Table S1.

### Isolation of AMs from BALF for *in vitro* experiment

For *in vitro* experiment, AMs were isolated as previously described 75. After WT mice were euthanized, BALF was collected by flushing the airways 10 times with 10 ml of sterile PBS (37 °C) containing 0.5% FBS (HyClone^TM^, Cytiva) and 2 mM EDTA using a 1 ml syringe. Cells were pelleted, subjected to red blood lysis, resuspended in RPMI culture medium (RPMI 1640 medium (Gibco) with 10% FBS, 1% penicillin/streptomycin, HEPES, GlutaMAX, and Sodium Pyruvate (Gibco)), and allowed to adhere to the specific culture wells (48-well plates for 10^6^ cells/ml, 96-well plates for 10^5^ cells/ml) for 3 h (37 °C, 5% CO_2_). After that, replaced the medium with fresh RPMI culture medium and proceeded *in vitro* treatment. Reagents and materials used are listed in Table S1.

### Bone marrow isolation and bone marrow-derived macrophages (BMDM) culture

Bone marrow cells were isolated by flushing femurs and tibia of 6- to 8-week-old WT mice. Isolated bone marrow was filtered with a 70 µM cell strainer, spun down at 500 g for 10 min at 4 °C, and red blood lysis was performed. Afterward, the resulting cells were cultured in DMEM medium (Gibco) supplemented with 20% mouse fibroblasts L929 (ATCC CCL-1) conditioned medium, 10% FBS, and 1% penicillin/streptomycin (Table S1). At day 4 and day 7, fresh medium with L929 cell conditioned medium was added. BMDMs were used on day 10 for *in vitro* experiment.

### Bulk RNA-sequencing

RNA was extracted from FACS-sorted AMs isolated from the BALF of PBS or Van-treated mice following LPS stimulation. Total RNA extraction was performed using REzol reagent (Protech) followed by purification with the Direct-zol RNA Microprep Kits (Zymo) (Table S1), according to the manufacturer's instructions. The purified RNA was used for the preparation of the sequencing library by TruSeq Stranded mRNA Library Prep Kit (Illumina, San Diego, CA, USA) following the manufacturer's recommendations. Briefly, mRNA was purified from total RNA (1 µg) by oligo(dT)-coupled magnetic beads and fragmented into small pieces under elevated temperature. The first-strand cDNA was synthesized using reverse transcriptase and random primers. After the generation of double-strand cDNA and adenylation on 3' ends of DNA fragments, the adaptors were ligated. The products were enriched with PCR and purified with AMPure XP system (Beckman Coulter, Beverly, USA). The libraries were qualified by Qsep400 System (Bioptic Inc., Taiwan) and quantified by Qubit 2.0 Fluorometer (Thermo Scientific, Waltham, MA, USA). The qualified libraries were then sequenced on an Illumina NovaSeq platform with 150 bp paired-end reads generated by Genomics, BioSci & Tech Co., New Taipei City, Taiwan.

### Lung histology

Lung tissues were harvested and fixed in 4% paraformaldehyde for 24 h, then dehydrated in 30%, 50%, and 70% ethanol for 10 min each, respectively. Tissue sections were prepared and stained with hematoxylin-eosin (H&E) to evaluate inflammation. Images were acquired using an Olympus CX31 microscope with 20× or 40× objectives (numerical aperture 0.4 and 0.65, respectively), and processed with CellSens imaging software.

### Neutrophil cell counting

The BALF was centrifuged at 500 g for 5 min to pellet the cells, and the supernatant was collected for cytokine measurement. Further, the cells were resuspended with 1 ml PBS, and 100 μl of the cell suspension was transferred to perform Cytospin at 1300 rpm for 5 min, immobilizing the cells onto glass microscope slides. The slides were then stained using the Diff-Quik Stain Kit (Sysmex) for cell identification under microscopy.

### ELISA

BALF or lung homogenates from mice, or culture supernatants from cells, were collected. ELISA was applied to measure the amounts of IL-1β (BioLegend), IL-17A (Invitrogen), and IL-22 (BioLegend) (Table S1) according to the manufacturer's instructions.

### RT-qPCR

Total RNA from lung tissue or cells was extracted by REzol reagent (Protech) and Direct-zol RNA Kits (Zymo) according to the manufacturer's instructions. Synthesis of cDNA was performed with a High-Capacity cDNA Reverse Transcription Kit (Thermo Fisher). Real time quantitative PCR was performed with the SYBR Green qPCR Master Mix (LabStar) on TOptical real-time PCR system (Biometra). The expression of target genes was normalized to expression of housekeeping gene *Gapdh*. All measurements were performed in duplicate. Reagents and materials used are listed in Table S1. Primer sequences are listed in Table S2.

### Immunoblot

The cells were incubated in lysis buffer containing protease inhibitor cocktail and subjected to sonication with a 10 s pulse and 10 s rest for 10 cycles. The total protein concentration was calculated using Micro BCA^TM^ Protein Assay Kit (Thermo Fisher). The equal amount of protein (12 μg/sample) loading with buffer mixture was boiled to denature at 95 °C for 5 min before the SDS-PAGE gel electrophoresis for protein separation. After electrophoresis, the proteins were wet-transferred to nitrocellulose membranes (Millipore), blocked with 5% BSA in 1×TBS containing 0.05% Tween-20 and then blotted for indicated proteins using the antibodies. Antibodies and reagents used are listed in Table S1.

### Immunofluorescence

The cells were fixed with 4% paraformaldehyde (PFA) at room temperature and methanol at 4 °C for 15 min, respectively. Further, the cells were blocked and permeabilized with blocking buffer (PBS containing 5% BSA and 0.3% TritonX-100) for 1 h, followed by sequential incubation with rabbit anti-GPR43 (bs-13536R, Bioss) overnight at 4 °C and DAPI for 1 min at room temperature. Fluorescence images were acquired using a laser-scanning confocal microscope (Olympus FV1200) equipped with 40× objectives (numerical aperture 0.6). Separate laser lines were used for excitation to prevent spectral overlap and cross-interference among fluorophores. Antibodies and reagents used are listed in Table S1.

### Statistical analysis

Statistical analyses were performed using Prism software (v9.5.1). For the comparisons between 2 groups, 2-tailed Student's t test for unpaired data was used. For multiple groups comparisons, one-way ANOVA were used. A p value of less than 0.05 was considered significant. All *in vivo* experiments were repeated at least twice and combined data are presented as mean ± SEM. All *in vitro* experiments were repeated independently 2-3 times and data are presented as mean ± SEM. Statistical details can be found in the figure legends.

## Supplementary Material

Supplementary figures and tables.

## Data Availability

16S rRNA-seq data generated in this study have been deposited at NCBI SRA under accession number PRJNA1345532. Bulk RNA sequencing data generated in this study have been deposited at NCBI GEO under accession number GSE309138. Published Single-cell RNA-seq data for lung cells are available at NCBI GEO under accession number GSE262927 (mouse) and GSE161382 (human). All data needed to evaluate the conclusions in the paper are present in the paper or the Supplementary Materials.

## Abbreviations

AM: Alveolar Macrophage
BALF: Bronchoalveolar Lavage Fluid
BMDM: Bone Marrow-Derived Macrophage
cAMP: Cyclic Adenosine Monophosphate
Clo: Clodronate
CM: Conditioned Medium
ELISA: Enzyme-linked Immunosorbent Assay
FACS: Fluorescence-activated Cell Sorting
FFAR: Free Fatty Acid Receptor
GF: Germ Free
GO: Gene Ontology
GPR: G Protein-coupled Receptor
GSEA: Gene Set Enrichment Analysis
HDAC: Histone Deacetylases
ILC: Innate Lymphoid Cell
IM: Interstitial Macrophage
KEGG: Kyoto Encyclopedia of Genes and Genomes
K.p.: *Klebsiella pneumoniae*
LC-MS: Liquid Chromatography-Mass Spectrometry
LPS: Lipopolysaccharide
MAPK: Mitogen-Activated Protein Kinase
MF: Molecular Functions
NF-κB: Nuclear Factor Kappa-light-chain-enhancer of activated B cells
NLRP3: NLR family pyrin domain containing 3
NMDS: Non-Metric Multidimensional Scaling
PFA: Paraformaldehyde
PKA: Protein Kinase A
RT-qPCR: Reverse-transcription Quantitative Polymerase Chain Reaction
SCFA: Short Chain Fatty Acid
SPF: Specific Pathogen Free
TLR: Toll-like Receptor
Van: Vancomycin
WT: Wild Type
