# Supplementary material for: Lung dysbiosis disrupts an FFAR2-mediated innate immune circuit against Klebsiella pneumoniae
Source: Theranostics. 2026 Mar 12;16(10):5348–69. doi: 10.7150/thno.131136 (PMC13080659; doi:10.7150/thno.131136)
Supplement: Supplementary file 1 — Supplementary figures and tables. [file thnov16p5348s1.pdf]

Supplementary Material for

Lung dysbiosis disrupts an FFAR2-mediated innate immune circuit against *Klebsiella pneumoniae*

Ting-Chieh Huang *et al.*

Corresponding author: Ya-Jen Chang, yajchang@ibms.sinica.edu.tw

The file includes:

Figure S1 to S5

Table S1 and S2

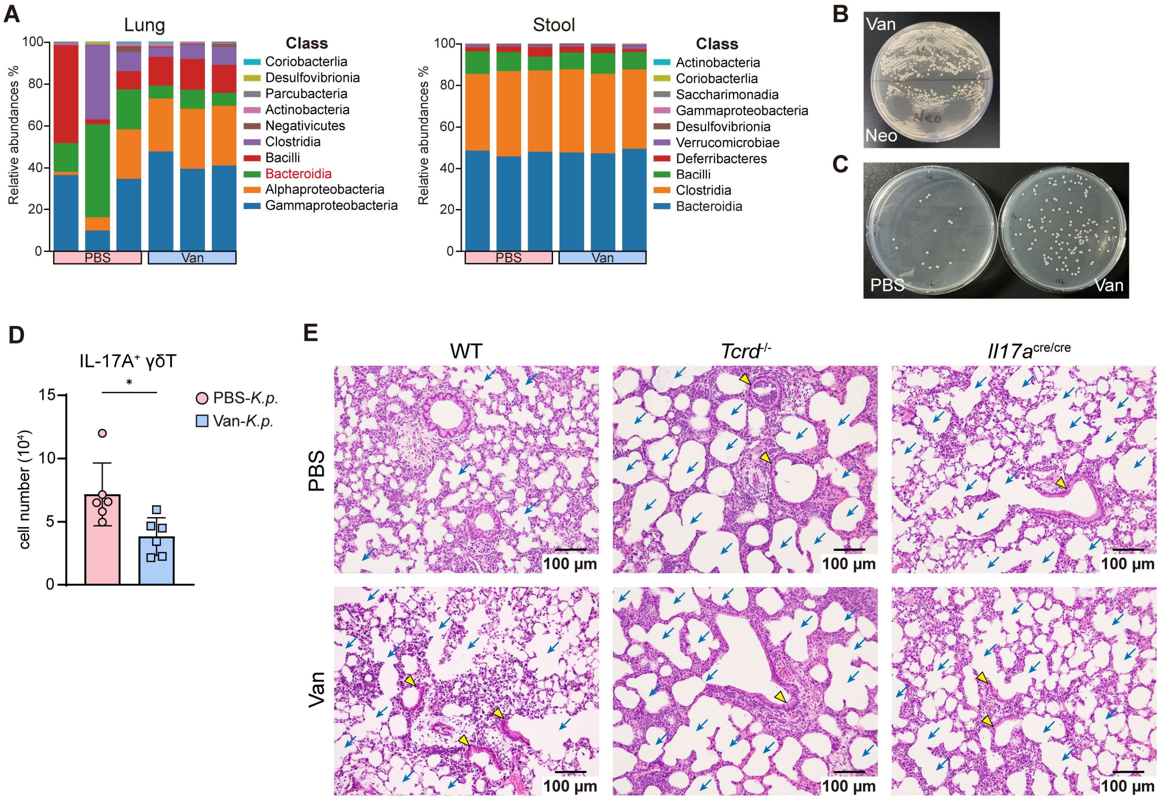

Figure S1. Vancomycin-induced lung dysbiosis weakens *K. pneumoniae* defense, an effect absents in *Tcrd*$^{-/-}$ and *Il17a*$^{cre/cre}$ mice. (**A**) Relative abundance of the lung (left) and gut (right) microbiota at class level between PBS and Van-treated group (n = 3). (**B**) Agar plate inoculated with *K. pneumoniae* following treatment of vancomycin (upper) or neomycin (below) (10 mg/ml). (**C**) Representative plate showing higher pulmonary *K. pneumoniae* bacterial burdens in Van group. (**D**) Absolute number of lung IL-17A^{+} γδ T cells (n = 6). (**E**) H&E staining of lung tissues from C57BL/6 WT mice, γδ T-deficient mice (*Tcrd*$^{-/-}$), or IL-17A-deficient mice (*Il17a*$^{cre/cre}$) following PBS or Van treatment and *K. pneumoniae* infection (bar, 100 μm). Data are representative of 1-2 independent experiments and values are shown as mean ± SEM; p-value were calculated by unpaired Student's t test (**D**). n.s. Not significant. *$p < 0.05$, **$p < 0.01$, ***$p < 0.001$, ****$p < 0.0001$.

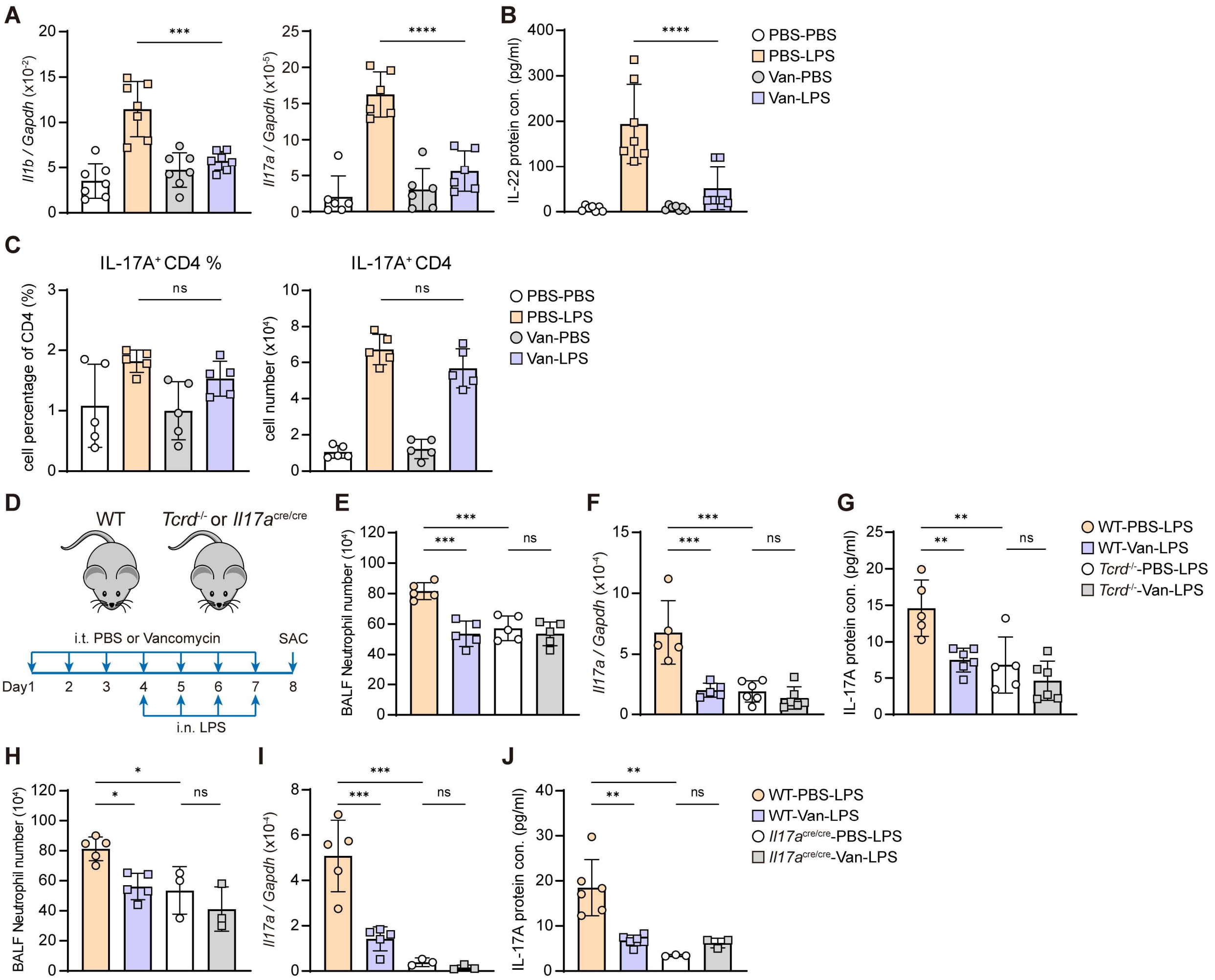

Figure S2. Lung dysbiosis-induced suppression of LPS-triggered acute inflammatory responses is not observed in *Tcrd*$^{-/-}$ and *Il17a*$^{cre/cre}$ mice. (A) mRNA expression of *Il1b* (left) and *Il17a* (right) in lung homogenates (n = 6-7). (**B**) Protein levels of IL-22 in BALF (n = 4-6). (**C**) Frequency quantification (left) and absolute number (right) of lung IL-17A$^+$CD4$^+$ T cells (Gating from CD4$^+$ T cells (CD45$^+$CD90.2$^+$CD4$^+$)) (n = 5). (**D**) Experimental design: Intratracheal vancomycin treatment was administrated to C57BL/6 WT mice, γδ T-deficient mice (*Tcrd*$^{-/-}$), or IL-17A-deficient mice (*Il17a*$^{cre/cre}$) for 7 days and intranasal LPS (2 μg/day) stimulation in the last 4 days, sacrificed one day after the last treatment. (**E-J**) The phenotype of inflammatory responses was elevated. BALF neutrophils numbers (**E**), mRNA expression (**F**) and protein levels (**G**) of IL-17A in lung lysates or BALF from WT compared to *Tcrd*$^{-/-}$ mice (n = 3-6). BALF neutrophil numbers (**H**), mRNA expression (**I**) and protein levels (**J**) of IL-17A in lung lysates or BALF from WT compared to *Il17a*$^{cre/cre}$ mice (n = 3-6). Data are representative of 2 independent experiments and values are shown as mean ± SEM; p-value were calculated by one-way ANOVA (**A-C**, **E-J**). n.s. Not significant. *p < 0.05, **p < 0.01, ***p < 0.001, ****p < 0.0001.

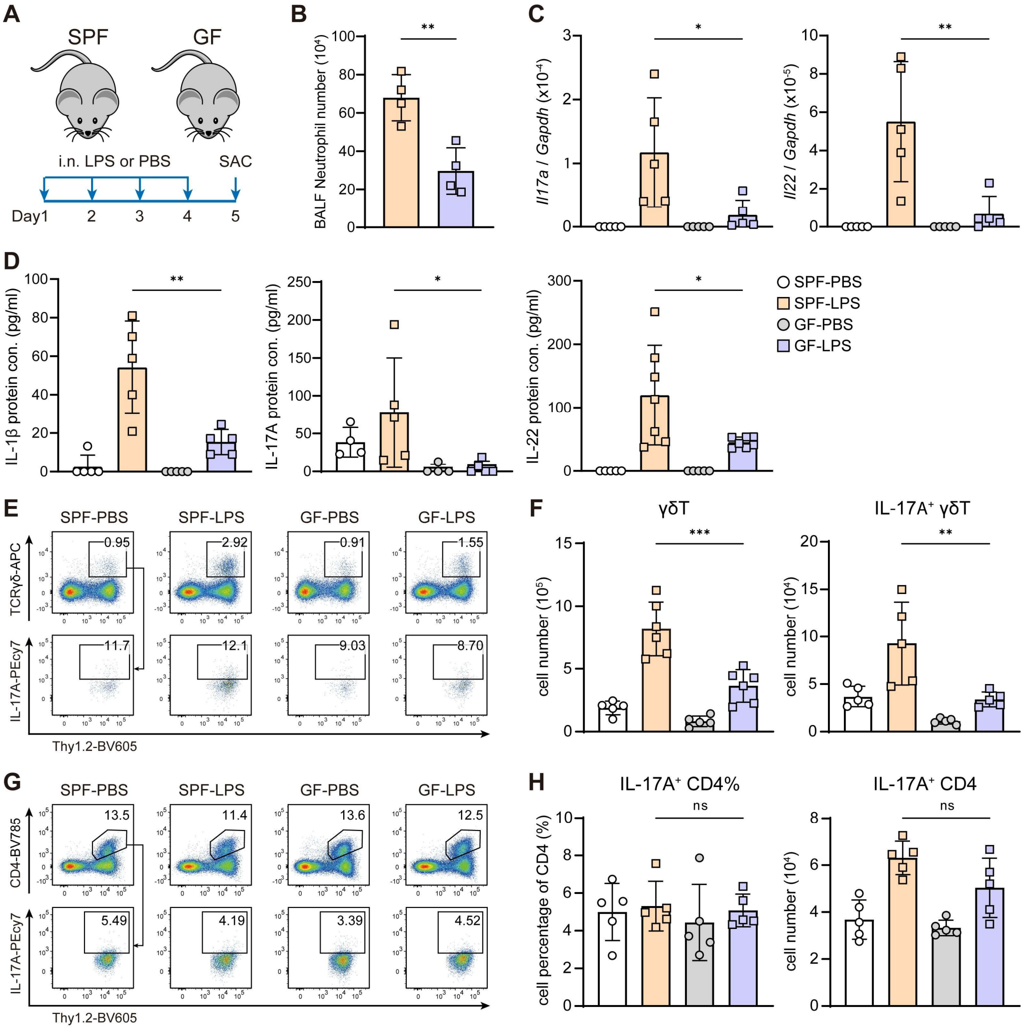

Figure S3. GF mice also exhibit lower LPS-induced acute inflammatory responses and γδ T cell activation compare with SPF mice. (**A**) Illustration of experimental model: GF or SPF mice were intranasally administrated with LPS (2 μg/day) for 4 days, and sacrificed at day 5. (**B-H**) The phenotype of inflammatory responses was elevated. (**B**) BALF neutrophils numbers (n = 5). (**C**) mRNA expression of *Il17a* (left) and *Il22* (right) in lung lysates (n = 5). (**D**) Protein levels of IL-1β (left), IL-17A (middle) and IL-22 (right) in BALF (n = 5-7). (**E**) Representative flow cytometry plots of IL-17A$^+$ γδ T cells (Gating from γδ T cells CD45$^+$CD90.2$^+$TCRγδ$^+$)). (**F**) Absolute number of γδ T cells (left) and IL-17A$^+$ γδ T cells (right) in the lung (n = 5-6). (**G**) Representative flow cytometry plots of IL-17A$^+$CD4$^+$ T cells (Gating from CD4$^+$ T cells (CD45$^+$CD90.2$^+$CD4$^+$)). (**H**) Frequency (left) and absolute number (right) of lung IL-17A$^+$CD4$^+$ T cells (n = 5). Data are representative of 2 independent experiments and values are shown as mean ± SEM; p-value were calculated by one-way ANOVA (**B-D**, **F**, **H**). n.s. Not significant. *p < 0.05, **p < 0.01, ***p < 0.001, ****p < 0.0001.

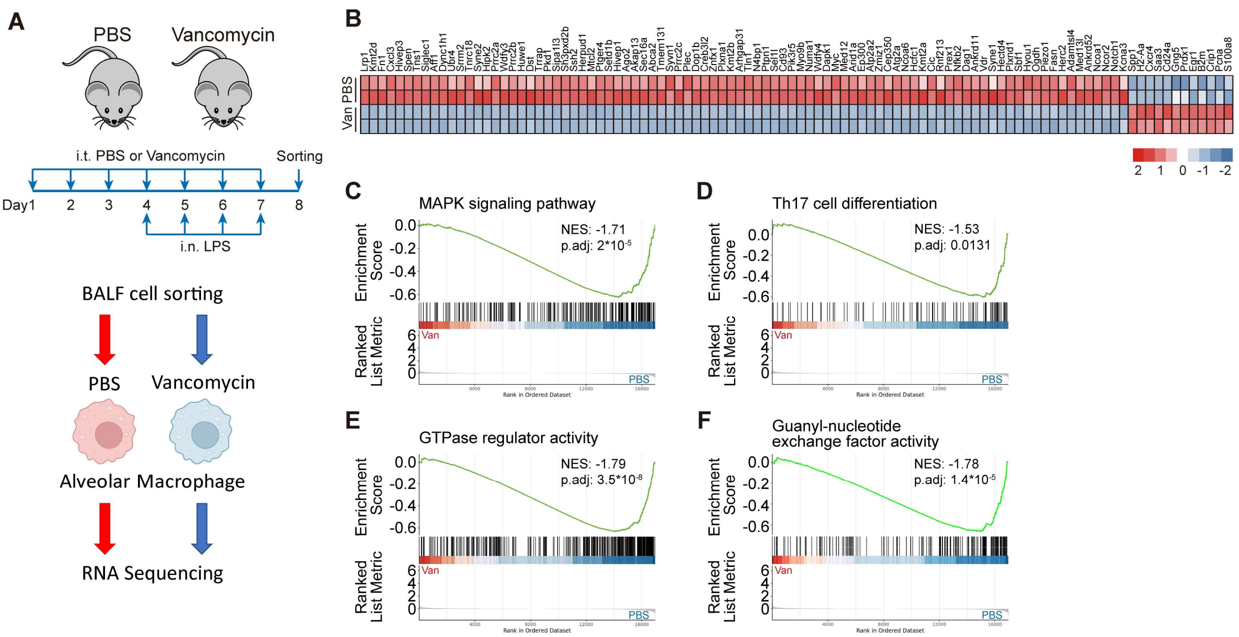

Figure S4. Intratracheal vancomycin-induced lung dysbiosis alters gene expression in AMs. (**A**) FACS-sorted AMs were isolated from the BALF of PBS- or Van-treated mice that had been previously administered intranasal LPS stimulation. The transcriptional profiling was evaluated by bulk RNA sequencing. (**B**) Heatmap of the top 50 selected significant differentially expressed genes (DEGs) in AMs from PBS or Vancomycin-treated group (z-score) (n = 2). (**C**-**F**) Barcode plots from GSEA of MAPK signaling pathway (**C**), Th17 cell differentiation (**D**), GTPase regulator activity (**E**), and Guanyl-nucleotide exchange factor activity (**F**) in AMs from Van-treated versus PBS-treated mice. Data are representative of 2 independent experiments and values are shown as mean ± SEM; p-value were calculated by one-sided Wilcoxon rank-sum test (**C**-**F**). n.s. Not significant. *$p < 0.05$, **$p < 0.01$, ***$p < 0.001$, ****$p < 0.0001$.

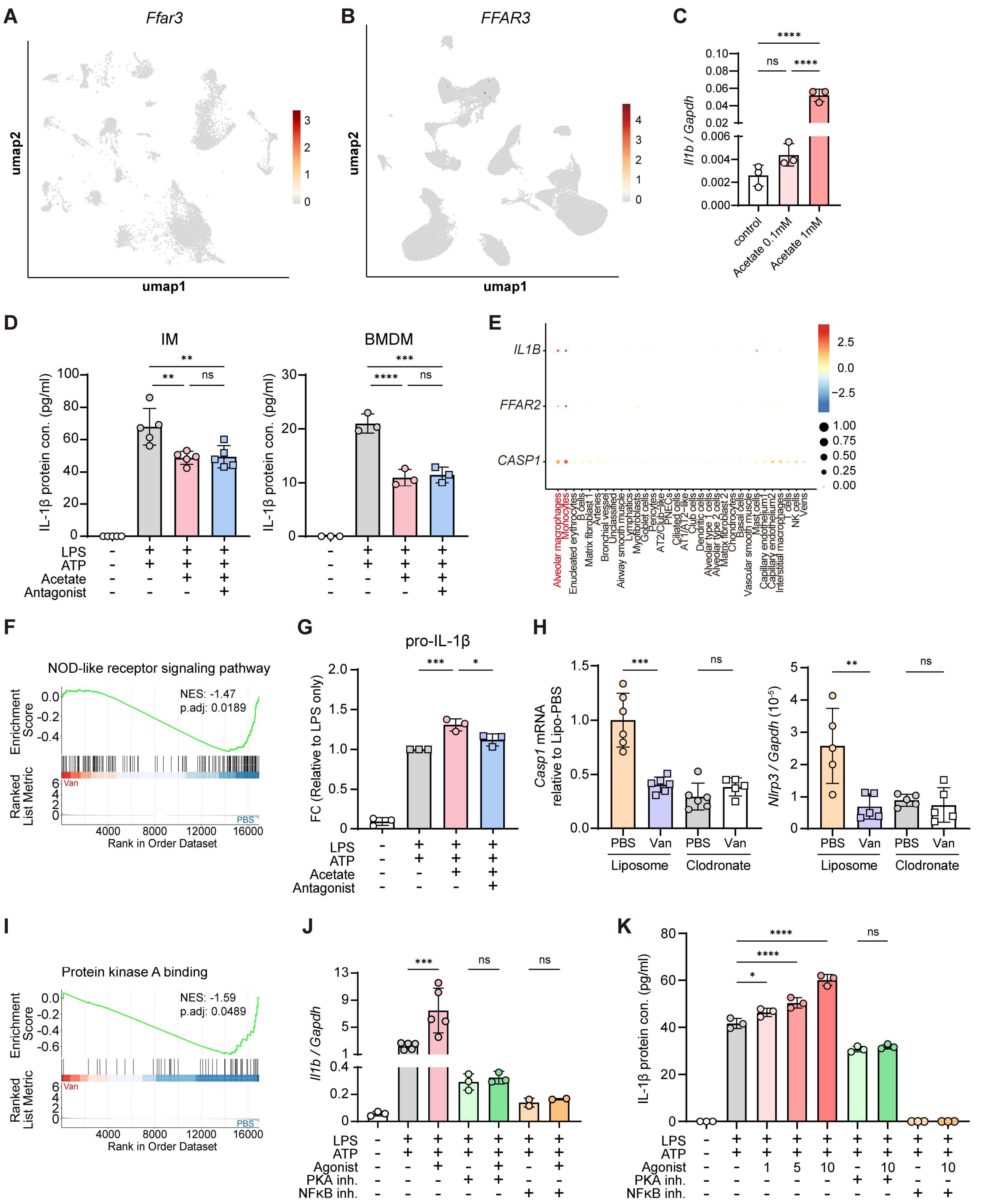

Figure S5. Acetate promotes IL-1β production via FFAR2-PKA-NF-κB axis in AMs, but not in IMs or BMDMs. (**A**, **B**) Feature plot illustrating the enrichment of FFAR3 in WT naïve murine lung (**A**) and health human lung (**B**), respectively. The scRNA-seq data were obtained and reanalyzed from GSE262927 and GSE161382. (**C**) mRNA expression of *Il1b* in sorted AMs (10^6 cells/ml) was evaluated by RT-qPCR following treatment with different concentration of acetate for 6 h. (**D**) Sorted IMs (10^5 cells/ml) (left) and cultured BMDMs (10^5 cells/ml) (right) were pre-incubated with or without FFAR2 antagonist (10 μM) for 30 min, followed by treatment with or without acetate (1 mM) for another 30 min. Subsequently, the

cells were stimulated with LPS (1 μg/ml) for 24 h, with ATP (2 mM) added in the last 30 min to induce inflammasome activation. The supernatants were then collected for IL-1β determination. (**E**) Bubble plot of total health human lung cells showing the expression levels of *IL1B*, *FFAR2* and *CASP1* across annotated cell types. Bubble size represents the percentage of cells expressing each gene; color intensity indicates mean expression level. The scRNA-seq data was obtained and reanalyzed from GSE161382. (**F**) Barcode plots from GSEA of NOD-like receptor activity in AMs from Van-treated versus PBS-treated mice. (**G**) The quantification for immunoblot analysis of pro-IL-1β in protein extracted from AMs. (**H**) mRNA expression of *Casp1* (left) and *Nlrp3* (right) in lung homogenates from the LPS stimulated mice, with or without intratracheal vancomycin treatment and intranasal clodronate administration for AMs depletion. (**I**) Barcode plots from GSEA of protein kinase A binding in AMs isolated from the BALF of Van-treated versus PBS-treated mice with LPS stimulation. (**J**, **K**) Sorted AMs were pre-incubated for 30 min with or without FFAR2 antagonist (10 μM), PKA inhibitor (10 μM) or NF-κB inhibitor (10 μM), followed by treatment with or without FFAR2 agonist (10 μM or indicated concentration (μM)) for an additional 30 min. Subsequently, the cells were stimulated with LPS (1 μg/ml) for 6 h (RT-qPCR (10^6 cells/ml)) or 24 h (ELISA (10^5 cells/ml)), with ATP (2 mM) added in the last 30 min to induce inflammasome activation. The cell lysates and supernatants were then collected for IL-1β determination by RT-qPCR (**J**) and ELISA (**K**). Data are representative of 2 independent experiments and values are shown as mean ± SEM; p-value were calculated by one-way ANOVA (**C**, **D**, **G**, **H**, **J**, **K**). n.s. Not significant. *p < 0.05, **p < 0.01, ***p < 0.001, ****p < 0.0001.

Table S1. Reagents and Materials

REAGENT OR RESOURCE	SOURCE	IDENTIFIER
Antibodies		
Caspase-1 Rabbit Polyclonal	GeneTex	Cat# GTX101322
CD11c APC Clone N418	BioLegend	Cat# 117309
CD11c FITC Clone N418	BioLegend	Cat# 117305
CD4 BV785 Clone GK1.5	BioLegend	Cat# 100453
CD45 PerCP/Cy5.5 Clone 30-F11	BioLegend	Cat# 103131
CD64 PE-Cy7 Clone X54-5/7.1	BioLegend	Cat# 139313
CD90.2 (Thy1.2) BV605 Clone 30-H12	BioLegend	Cat# 105343
F4/80 FITC Clone BM8	BioLegend	Cat# 123107
FFAR2 (GPR43) Rabbit Polyclonal	Bioss	Cat# bs-13536R
Goat anti-Mouse IgG (H+L) Secondary Antibody, HRP	Thermo Fisher	Cat# 31430
Goat anti-Rabbit IgG (H+L) Secondary Antibody, HRP	Thermo Fisher	Cat# 31460
IL-17A PE-Cy7 Clone TC11-18H10.1	BioLegend	Cat# 506921
IL-1β PE Clone NJTEN3	Invitrogen	Cat# 12-7114-82
IL-1β Rabbit Polyclonal	GeneTex	Cat# GTX74034
Ly6G BV421 Clone 1A8	BioLegend	Cat# 127627
Ly6G BV605 Clone 1A8	BioLegend	Cat# 127639
NF-κB p65 Rabbit Clone D14E12	Cell Signaling	Cat# 8242
NLRP3 Mouse Clone Cryo-2	AdipoGen	Cat# AG-20B-014-C100
p-NF-κB p65 (Ser536) PE Clone 93H1	Cell Signaling	Cat# 5733
p-NF-κB p65 (Ser536) Rabbit Clone 93H1	Cell Signaling	Cat# 3033
Siglec-F BV421 Clone S17007L	BioLegend	Cat# 155509
Siglec-F PE Clone S17007L	BioLegend	Cat# 155505
TCR γ/δ APC Clone GL3	BioLegend	Cat# 118115

TCR γ/δ BV421 Clone GL3	BioLegend	Cat# 118119
β-actin Clone BA3R	Invitrogen	Cat# MA5-15739
Bacterial strains		
Klebsiella pneumoniae NCTC 9633	ATCC	ATCC 13883
Chemicals, peptides, and recombinant proteins		
ATP disodium salt hydrate	Sigma-Aldrich	Cat# A2383
EDTA 0.5M	Corning	Cat# 46-034-Cl
Fetal bovine serum (FBS)	HyClone™, Cytiva	Cat# SH30088
FFAR2 agonist (TUG-1375)	MCE	Cat# HY-112813
FFAR2 antagonist (GLPG0974)	Tocris	Cat# 5621
Fixable Viability Dye eFluor™ 780	Invitrogen	Cat# 65-0865-14
GlutaMAX	Gibco	Cat# 35050061
GolgiStop	BD	Cat# 554724
HEPES (1M)	Gibco	Cat# 15630130
IL-1 receptor antagonist (Anakinra)	Sobi Kineret®	CAS# 143090-92-0
Intracellular Fixation & Permeabilization Buffer Set	Invitrogen	Cat# 88-8824-00
Ionomycin from *Streptomyces conglobatus*	Sigma-Aldrich	Cat# I9657
Klebsiella ChromoSelect Selective Agar Base	Merck	Cat# 90925
Klebsiella Selective Supplement (Carbenicillin)	Merck	Cat# 15821
Lipopolysaccharides from *Klebsiella pneumoniae*	Sigma-Aldrich	Cat# L4268
NF-κB inhibitor (BAY 11-7082)	MCE	Cat# HY-13453
PBS (1x)	Leinco	Cat# P364
Percoll	Cytiva	Cat # 17089101
Phorbol 12-myristate 13-acetate (PMA)	Sigma-Aldrich	Cat# P8139
PKA inhibitor (H 89 2HCl)	Selleckchem	Cat# S1582
Polypropylene Microvials-2 ml	BioSpec	Cat# 10831

RPMI 1640	Gibco	Cat# 11875093-500mL
Sodium acetate	Sigma-Aldrich	Cat# S8750
Sodium Pyruvate (100mM)	Gibco	Cat# 11360070-100mL
Standard Macrophage Depletion Kit (Clodrosome® + Encapsome®)	Encapsula Nanosciences	Cat# CLD-8901
Tryptic Soy Broth- Dehydrated Culture Media	Merck	Cat# 22092
Vancomycin hydrochloride	Sigma-Aldrich	Cat# V2002
Zirconia/Silica Beads-1 mm	BioSpec	Cat# 11079110z
Critical commercial assays		
Direct-zol RNA Miniprep/Microprep Kits	Zymo	Cat# R2050/R2060
ELISA MAX™ Deluxe Set Mouse IL-1β	BioLegend	Cat# 432604
ELISA MAX™ Deluxe Set Mouse IL-22	BioLegend	Cat# 436304
High-Capacity cDNA Reverse Transcription Kit	Thermo Fisher	Cat# 4374967
Micro BCA™ Protein Assay Kit	Thermo Fisher	Cat# 23235
Mouse IL-17A (homodimer) Uncoated ELISA Kit	Invitrogen	Cat# 88-7371-88
Pierce™ BCA Protein Assay Kits	Thermo Fisher	Cat# A55865
QIAamp Fast DNA Stool Mini Kit	QIAGEN	Cat# 51604
Deposited data		
Raw data files for 16S rRNA sequencing of lung and stool	This paper	PRJNA1345532
Raw data files for bulk RNA sequencing of AMs	This paper	GSE309138
Experimental models: Mouse strains		
B6.129P2-*Tcrd*^{tm1Mom}/J (*Tcrd*$^{-/-}$)	Jackson Laboratories	Strain# 002120
B6(Cg)-*Tlr4*$^{tm1.2Karp}$/J (*Tlr4*$^{-/-}$)	Jackson Laboratories	Strain# 029015
C57BL/6J	National Laboratory Animal Center (Taipei, Taiwan)	N/A

Ffar2^{flox}	This paper	N/A
Germ-Free	National Laboratory Animal Center (Taipei, Taiwan)	N/A
Il17a^{cre}	Dr. Jr-We Shui	N/A
LysM^{cre}	Dr. Jr-We Shui	N/A
Oligonucleotides		
qPCR primers, see Table S2	This paper	N/A
Software and algorithms		
BioRender	BioRender Company	https://www.BioRender.com/
FlowJo (v10)	TreeStar	https://www.flowjo.com/solutions/flowjo/
GraphPad Prism 9	GraphPad	https://www.graphpad.com/features
Illustrator	Adobe	https://www.adobe.com/
ImageJ software	ImageJ	https://imagej.net/ij/
R (v3.5.3)	R Project	https://www.r-project.org/

Table S2. Primers used for qPCR and 16S rRNA sequencing

Species	Primer Name	Primer Sequence (5' to 3')		Purpose
Mouse	*Gapdh*	Forward	AGGTCGGTGTGAACGGATTTG	qPCR
		Reverse	TGTAGACCATGTAGTTGAGGTCA	
	Tjp1	Forward	ACAGGCCATTACGAGCCTCT	
		Reverse	GGAGGCTGTGGTTTGGTAGC	
	Il1b	Forward	GAAATGCCACCTTTTGACAGTG	
		Reverse	CTGGATGCTCTCATCAGGACA	
	Il17a	Forward	CAGACTACCTCAACCGTTCCAC	
		Reverse	TCCAGCTTTCCCTCCGCATTGA	
	Il22	Forward	TCGTCAACCGCACCTTTATG	
		Reverse	GCCGGACATCTGTGTTGTTATC	
	Casp1	Forward	ACAAGGCACGGGACCTATG	
		Reverse	TCCCAGTCAGTCCTGGAAATG	
	Nlrp3	Forward	ATTACCCGCCCGAGAAAGG	
		Reverse	TCGCAGCAAAGATCCACACAG	
	Ffar2	Forward	CTTGATCCTCACGGCCTACAT	
		Reverse	CCAGGGTCAGATTAAGCAGGAG	
Bacteria	16S rRNA V3-V4 region 340F	Forward	ACTCCTACGGGAGGCAGCAGT	
	16S rRNA V3-V4 region 514R	Reverse	ATTACCG CGGCTGCTGGC	
	16S rRNA V3-V4 region 341F	Forward	CCTACGGGNGGCWGCAG	16S rRNA Amplicon
	16S rRNA V3-V4 region 805R	Reverse	GACTACHVGGGTATCTAATCC	
